# Mac-1 blockade impedes adhesion-dependent neutrophil extracellular trap formation and ameliorates lung injury in LPS-induced sepsis

**DOI:** 10.3389/fimmu.2025.1548913

**Published:** 2025-03-28

**Authors:** Jinhua Fang, Hongguang Ding, Jiaqi Huang, Wang Liu, Tiantian Hong, Junxian Yang, Zhiwei Wu, Zhuo Li, Shiying Zhang, Peimin Liu, Ying Fang, Jianhua Wu, Xin Li, Jiangguo Lin

**Affiliations:** ^1^ Medical Research Institute, Guangdong Provincial People’s Hospital (Guangdong Academy of Medical Sciences), Southern Medical University, Guangzhou, China; ^2^ Department of Emergency Medicine, Guangdong Provincial People’s Hospital (Guangdong Academy of Medical Sciences), Southern Medical University, Guangzhou, China; ^3^ Institute of Biomechanics/School of Bioscience and Bioengineering, South China University of Technology, Guangzhou, China; ^4^ Department of Critical Care Medicine, Guangdong Provincial People’s Hospital (Guangdong Academy of Medical Sciences), Southern Medical University, Guangzhou, China; ^5^ Department of Nephrology, Guangdong Provincial People’s Hospital (Guangdong Academy of Medical Sciences), Southern Medical University, Guangzhou, China; ^6^ School of Medicine, South China University of Technology, Guangzhou, China

**Keywords:** sepsis, neutrophil extracellular traps, integrin Mac-1, endothelial cells, peptidylarginine deiminase 4

## Abstract

**Background:**

Sepsis is a common critical condition that can lead to multiple organ injury. Sepsis-induced acute respiratory distress syndrome (ARDS) is frequently an important cause of poor prognosis and is associated with high mortality rates, despite existing therapeutic interventions. Neutrophil infiltration and extracellular traps (NET) are implicated in acute lung injury (ALI) and ARDS following sepsis. As circulating neutrophils infiltrate infected tissues, they come into direct contact with vascular endothelial cells (ECs). Although the ability of NETs to induce endothelial damage is well established, the specific role of direct EC-neutrophil interactions in NET formation and lung injury during sepsis is not fully understood.

**Methods:**

In this study, NET formation was assessed when neutrophils were co-culture with ECs or separated from them and stimulated with phorbol 12-myristate 13-acetate (PMA), lipopolysaccharide (LPS), lipoteichoic acid (LTA), or septic plasma.

**Results:**

We found that adhesion of neutrophils on ECs is critical in NET formation in response to LPS, LTA, or septic plasma in vitro. Blocking the macrophage-1 antigen (Mac-1) impeded NET formation, while inhibiting P-selectin glycoprotein ligand-1 (PSGL-1) or leukocyte function-associated antigen-1 (LFA-1) did not. This adhesion-dependent NET formation was reliant on the influx of extracellular calcium and peptidylarginine deiminase 4 (PAD4)-mediated citrullination of histone H3. However, Mac-1 blockade did not alter calcium influx. In a murine model of LPS-induced sepsis, Mac-1 blockade reduced NET release, lowered inflammatory cytokine levels, mitigated endothelial damage, and attenuated lung injury.

**Conclusion:**

Our findings offer insights into the critical role of EC-neutrophil direct contact in NET formation during sepsis and propose Mac-1 as a potential therapeutic target.

## 1 Introduction

Severe sepsis is characterized by systemic inflammation in response to infection, often leading to multi-organ injury (1). The lungs are typically the first and most susceptible organ affected, with acute respiratory distress syndrome (ARDS) being the most common and lethal complication (2). Despite advancements, the incidence of sepsis-induced ARDS continues to rise, representing a significant clinical challenge with a mortality rate exceeding 25%, even with effective antimicrobial therapy (3).

Neutrophils are pivotal effector cells in sepsis (4), with their accumulation in lung interstitial and alveolar space being a hallmark of ARDS (5, 6). Early in the immune response, neutrophils migrate to the inflammation site, engaging pathogens through phagocytosis, degranulation, and releasing neutrophil extracellular traps (NETs) (7, 8). NETs are a meshwork of decondensed chromatin decorated with antimicrobial proteins, including histones, neutrophil elastase (NE), and myeloperoxidase (MPO) (9). A large number of neutrophil infiltrates and NETs are found in the lung tissue of patients with sepsis, and the production of excess NETs has been shown to promote inflammation and damage tissue (10). NE released from activated neutrophils damages the lung endothelium, significantly contributing to ARDS (11). During lipopolysaccharide (LPS)-induced acute lung injury (ALI), NETs have been shown to directly cause organ damage and amplify inflammatory response, marked by leukocyte accumulation, diffuse alveolar injury, and cytokine release (12). Thus, neutrophil infiltration and NET formation are crucial in ARDS pathogenesis during sepsis.

Activated platelet-mediated neutrophil recruitment and NET formation are well-documented (13–15). Toll-like receptor 4 (TLR4)-dependent platelet-neutrophil interactions induce NET production to trap bacteria in septic blood (16). Activated platelets present high mobility group box 1 (HMGB1) to neutrophils to promote NET formation (17, 18). Platelet-derived exosomes containing HMGB1 and/or miR-15b-5p and miR-378a-3p promote NET generation during septic shock (19). In contrast, endothelial cell (EC)-induced NET formation is less studied, although NETs and their components damaging EC are well documented (20, 21). Sepsis shifts ECs to a proapoptotic, proinflammatory, adhesive, and procoagulant phenotype (22). Activated ECs partly induce NET formation through interleukin 8 (IL-8) (23) and Exosome-encapsulated miR-505 from oxidized low-density lipoprotein-treated vascular ECs (24). Recruitment of neutrophils from blood vessels into infected tissue is vital in sepsis, involving a sequence of cellular events, including tethering, rolling, slow rolling, firm adhesion, crawling, and migration (25). In this process, neutrophils direct contact with ECs through adhesion molecules on both sides (26). Although EC-mediated neutrophil infiltration into lung tissue has been well studied, whether the direct EC-neutrophil interactions prompt NET formation in sepsis remains unclear.

Adhesion molecules, such as P-selectin glycoprotein ligand-1 (PSGL-1) and integrins, are crucial for neutrophil-EC interaction (27–30). PSGL-1 on neutrophils interacting with selectins on EC mediates neutrophil tethering and rolling. PSGL-1-P-selecitn interaction has been reported to induce NET formation in a peptidyl arginine deiminase 4 (PAD4)-dependent manner in acute pancreatitis (31). However, low PSGL-1 expressing neutrophils from active systemic lupus erythematosus patients are more susceptible to generating NETs (32). Neutrophils mainly express β_2_-integrins (CD18), including leukocyte function-associated antigen-1 (LFA-1, CD11a/CD18) and macrophage-1 antigen (Mac-1, CD11b/CD18). During sepsis, activated ECs upregulate the expression of adhesion molecules, including the β2-integrin ligand intercellular adhesion molecule-1 (ICAM-1) (33). ICAM-1-β_2_-integrin interactions mediate the recruitment of neutrophils to the sites of inflammation. When neutrophils adhere to the endothelium, LFA-1 mediates neutrophil slow rolling and firm adhesion, while Mac-1 mediates neutrophil crawling and migration (30, 34). It has been reported that LFA-1 and Mac-1 are implicated in NET formation. Neutrophil-specific knockdown of β_2_-integrins impairs NET formation (28). Mac-1 has been reported to mediate NET formation induced by immobilized immune complexes (35), Aspergillus fumigatus (36), Candida albicans and immobilized beta-glucan (37, 38), hantavirus (39), and activated platelets (40). Neutralization of LFA-1 reduced NET formation induced by LPS-stimulated platelets (41). Therefore, the role and mechanism of adhesion molecules in EC-neutrophil-mediated NET formation in sepsis await investigation.

In this study, we demonstrated that neutrophils stably adhering to an EC layer or ICAM-1 coated surface generated NETs in response to LPS, lipoteichoic acid (LTA), phorbol 12-myristate 13-acetate (PMA), or septic patient plasma. Mac-1 blockade impaired NET formation *in vivo* and *in vitro* and ameliorated lung injury in a model of LPS-induced sepsis.

## 2 Materials and methods

### 2.1 Reagents

Histopaque 1119, PMA, LPS, LTA, NADPH oxidase inhibitor DPI, and endoplasmic reticulum Ca^2+^ inhibitor 2-APB were obtained from Sigma-Aldrich (St Louis, MO, USA). ICAM-1/CD54 was purchased from R&D (Minneapolis, MN, USA). Recombinant Human TNF-α was from Novoprotein (Suzhou, China). Sytox green and Hoechst33342 were from InvitrogenTM (Carlsbad, CA, USA). Anti-histone H3 (citrulline R2 + R8 + R17) antibody, anti-human neutrophil elastase antibody, and Alexa Fluor 594 secondary antibody were obtained from Abcam (Cambridge, UK). Anti-human CD11a antibody (Clone: HI111), mouse IgG1, κ Isotype ctrl antibody (Clone: MOPC-21), anti-mouse/human CD11b antibody (Clone: M1/70), and Rat IgG2b, κ Isotype ctrl antibody (Clone: RTK4530), and Alexa Fluor 647 anti-CD66 antibody were purchased from Biolegend (San Diego, CA, USA). PAD4 inhibitor GSK484 was from Cayman Chemical (Ann Arbor, MI, USA). Anti-human CD11b antibody CBRM1/5 was purchased from Santa Cruz (Dallas, TX, USA). DNase I was from Roche (Basel, Switzerland). Anti-myeloperoxidase mouse mAb, Cy3 conjugated Goat anti-Rabbit IgG, Alexa Fluor 488-conjugated Goat anti-mouse IgG, DAPI, and anti-fluorescence quenching sealing tablets were from Servicebio (Wuhan, China). Quant-iT PicoGreen dsDNA Assay Kit was from Thermo Fisher Scientific (Waltham, MA, USA). OriCell^®^ human umbilical vein endothelial cell complete culture medium was from Cyagen (Guangzhou, China).

### 2.2 Isolation of human neutrophils and plasma samples

Human peripheral blood samples of both septic patients and healthy donors were obtained from Guangdong Provincial People’s Hospital. This study was approved by the ethical committee of Guangdong Provincial People’s Hospital (KY2020-051-02), in conformity with the Helsinki Declaration. All human participants gave written informed consent. Blood was gently layered in a 1:1 ratio on top of Histopaque 1119 and centrifuged at 700 × g for 30 min. Then, the transparent third and pink fourth layers containing neutrophils were collected and washed with PBS. Red cell lysis buffer was employed to remove red blood cells from the collected neutrophil layer. After washing with the PBS, neutrophils were resuspended in RPMI-1640 containing 10% FBS medium (>95% purity by flow cytometry, no visible activation by microscopy).

A total of 20 septic patients and 20 healthy donors were enrolled in this study. Patient cohort baseline characteristics were shown in **Table 1**. Plasma samples were isolated by density gradient centrifugation from peripheral blood. In brief, blood was centrifuged at 200 × g for 10 min. The platelet-rich plasma was collected and centrifuged again at 2000 × g for 20 min to remove platelets. The platelet-poor plasma was stored at -80°C ready to use.

**Table 1:** Demographic information and hematological parameters of sepsis patients and healthy volunteers.

Characteristic	Sepsis patients (n = 20)	Healthy volunteers (n = 20)	*P* value
Male: Female	10:10	10:10	>0.99
Age (Y)	56.1 ± 3.0	50.8 ± 2.5	0.18
^†^CRP (mg/L)	140.8 ± 17.3	N/A	–
^‡^PCT (ng/mL)	65.9 ± 18.6	N/A	–
D-dimer (ng/mL)	8323.7 ± 1443.3	N/A	–
^§^SOFA Score	7.7 ± 0.8	N/A	–
^¶^APACHE II	18.0 ± 1.3	N/A	–
Primary pathogen, n (%)			
Gram -ve bacteria	9 (45%)	N/A	–
Gram +ve bacteria	7 (35%)	N/A	–
Culture negative	4 (20%)	N/A	–

^†^CRP, C-reactive protein; ^‡^PCT, procalcitonin; ^§^SOFA Score, sequential organ failure assessment score; ^¶^APACHE II, acute physiology and chronic health evaluation II. Data are present as mean ± SEM.

### 2.3 Inhibitors and neutralizing monoclonal antibodies

In some experiments, neutrophils were pretreated for 30 min with the indicated inhibitors or neutralizing antibodies: anti-CD11a I-domain antibody (clone HI111, 1:100), anti-CD11b lectin domain antibody (clone M1/70, 1:100 dilution), anti-CD11b I-domain antibody (clone CBRM1/5, 1:100 dilution), DPI (NADPH oxidase inhibitor, 10 μM), GSK484 (PAD4 inhibitor, 10 μM), and 2-APB (endoplasmic reticulum Ca^2+^ inhibitor, 100 μM). Isotype control antibodies served as control.

### 2.4 NET formation on the EC layer

Human umbilical vein ECs (HUVECs, ATCC Cat# CRL-1730) were cultured in OriCell human umbilical vein endothelial cell complete culture medium. During NET induction experiments, HUVECs were cultured in RPMI-1640 containing 10% FBS. For endothelial activation, HUVEC were treated with 10 ng/mL TNF-α for 24 hours and then washed with warmed FBS prior to experimentation. In the first set of experiments, HUVECs were inoculated in 96-well black culture plates (Corning, New York, USA). When the cell fusion was greater than 90%, neutrophils (4×10^5^ cells/well) were added to co-cultured with HUVECs for 30 min. After removing the nonadherent neutrophils, the cells were stimulated with PMA (100 ng/mL), LPS (5 μg/mL), or LTA (40 μg/mL) for 4 h. In the second set, HUVECs were cultured in a Millicell cell chamber with a pore size of 0.4 μm (Millipore, Temecula, CA, USA), preventing cells from passing through, placed in a 24-well plate (NEST, Wuxi, China) in culture media containing neutrophils (2×10^6^ cells/well). PMA, LPS, or LTA were employed to stimulate cells. extracellular DNA was stained with a membrane-impermeable DNA binding dye, Sytox green. Alexa Fluor 647 anti-CD66 antibody (1:50 dilution) was used to identify neutrophils. NET formation was detected using a multifunctional microporous plate detector (Spark, TECAN, Switzerland).

### 2.5 NET formation on the ICAM-1 layer and quantification

Purified neutrophils (4×10^5^ cells/well) were seeded on the glass surface (8 well µ-Slides, Ibidi, Munich, Germany) coated with ICAM-1 (100 μg/mL) and incubated at 37°C, 5% CO_2_ for 30 min. After removing nonadherent neutrophils, PMA (100 ng/mL), LPS (100 ng/mL), LTA (4 μg/mL), and plasma from healthy donors or from septic patients (20%) were added to stimulate adherent neutrophils for 4 h. For inhibition and blocking experiments, antibody- or inhibitor-treated neutrophils were used. In some experiments, 100 U/mL DNase1 was added to digest extracellular DNA for 20 min. To determine the role of extracellular Ca^2+^, EGTA at a final concentration of 10 mM was added to chelate extracellular Ca^2+^. Extracellular DNA was stained with Sytox green and citrullination histone 3 (CitH3) was stained with anti-Histone H3 (citrulline R2 + R8 + R17) antibody (1:200 dilution) and Alexa Fluor 594 secondary antibody (1:200 dilution). For NET quantification, 10-15 images of each experiment at 60× were randomly selected by fluorescence microscopy (Ti2-U, Nikon, Tokyo, Japan). All images were analyzed using ImageJ software (National Institute of Health, Bethesda, MD, USA). Sytox green-positive and CitH3-positive objects were selected and the area and fluorescence intensity were measured. To exclude the influence of cell number, the measured area and fluorescence intensity were divided by the total number of the cells, as counted in bright-field images. The results were presented as fold changes normalized to the control group.

### 2.6 Western blot analysis

To assess CitH3 protein levels, neutrophils (1 × 10^6^ cells) were washed with cold PBS and lysed with RIPA buffer (1 mM PMSF) following stimulation. The cell lysate was centrifuged at 17000 × g for 20 min at 4°C. The resulting supernatant was subjected to SDS-polyacrylamide gel electrophoresis and transferred for Western blot analysis. Rabbit Polyclonal Histone H3 (Novus, 1:500 dilution) and recombinant anti-GAPDH antibody (Servicebio, 1:3000 dilution) were applied as primary antibodies. Goat anti-rabbit IgG HRP (Affinity, 1:5000 dilution) was used as the secondary antibody. Chemiluminescence signals were detected using the ChampChemi 910 imaging system (Sinsage, Beijing, China).

### 2.7 Flow cytometry

To measure the formation of NETs from suspended neutrophils in response to stimulants, flow cytometry was used. Neutrophils were incubated with or without soluble ICAM-1 (200 μg/mL) and stimulated with PMA (100 ng/mL), LPS (100 ng/mL), or LTA (4 μg/mL) for 4 h at 37°C. After washing three times with PBS containing 2% BSA, neutrophils were incubated with Sytox green for 15 min. The ratio of NET-forming neutrophils was detected using a Beckman Cytoflex flow cytometer (Beckman Coulter, Indianapolis, IN, USA).

### 2.8 Animal model

Male C57Bl/6J mice, aged 8-10 weeks, were purchased from Zhuhai BesTest Bio-Tech Co,.Ltd (Zhuhai, China). Mice were separated into 4 experimental groups: saline control group, LPS-treated group, LPS-treated group with M1/70, and LPS-treated group with Isotype IgG2b. 20 mg/kg of LPS from Escherichia coli 0111: B4 was administered intra-peritoneum (i.p) at 30 min after the tail vein injection by saline or therapeutic reagents (100 μg M1/70, or 100 μg Isotype IgG2b) (42). The mice were sacrificed at 8 hours after sepsis induction. Plasma and lung samples were collected and stored in the freezer at −80°C for further analysis. The wet weight of the left lung was measured using an electronic scale. The lung was then desiccated to determine its dry weight. Water content was calculated as the wet/dry weight ratio. The right lung was lavaged with 0.5 ml of cold PBS per instillation, totaling 1.5 ml of bronchoalveolar lavage fluid (BALF) collected from each mouse. BALF was analyzed to determine total protein levels and cell counts. To assess whether treated with Mac-1 antibody affects survival, lethal dose (30 mg/kg) of LPS were i.p administered to the mice 30 min after injection of M1/70 or Isotype IgG2b (100 μg). Survival was monitored up to 96 hours. This study was ethically approved by the Institutional Animal Care and Use Committee of Guangdong Provincial People’s Hospital.

### 2.9 Quantification of DNA and protein levels in plasma and BALF

Cell-free DNA from plasma was quantified by Quant-iT PicoGreen dsDNA Assay Kit (Thermo Fisher Scientific, USA) according to the manufacturer’s instructions. Briefly, 200 μL samples were mixed with 200 μL PicoGreen to label DNA. Fluorescence was monitored with a multifunctional microporous plate detector (Spark, TECAN, Switzerland). DNA concentration was calculated based on a standard curve of known concentrations of DNA. The amount of circulating CitH3-DNA complex was measured using a modified capture ELISA technique. The plasma containing anti-DNA-POD antibody was incubated in 96-well plates precoated with CitH3 antibodies and color-developed with ABST solution. The absorbance of 405 nm was measured. Levels of TNF-α, IL-1β, and IL-6 in mouse plasma and BALF were determined using a commercially available enzyme-linked immunosorbent assay (ELISA) kit, following the manufacturer’s instructions.

### 2.10 Histology and immunohistochemistry

For lung injury analysis, the histological index was calculated. Histological index included vascular congestion, leukocyte infiltration, necrosis, and alveolar wall thickness. Each item was divided into four grades ranging from 0 to 3 (0 = normal; 1 = mild; 2 = moderate; 3 = severe), and then a total score was calculated. The scoring was conducted in a double-blind manner. The alveolar septa thickness was measured by analyzing randomly selected four fields of view for each slide using an image analysis system (Image-Pro Plus software). Briefly, alveolar spaces were filled with black, while alveolar septa were filled with white. The white areas were measured, which represent the areas of alveolar septa. Then the white areas were thinned into lines, the lengths of which were measured. Alveolar septa thickness = areas of alveolar septa/lengths of alveolar septa.

For immunohistochemistry, slides were incubated with primary antibodies (anti-CitH3 antibody and anti-MPO antibody) and secondary antibodies (Cy3 conjugated Goat anti-Rabbit IgG (1:300 dilution) and Alexa Fluor 488-conjugated Goat anti-Mouse IgG (1:400 dilution)). Slides were mounted on glass slides in anti-fluorescence quenching sealing tablets, and coverslips were applied. All the images were obtained with fluorescence microscopy (Ti2-U, Nikon, Tokyo, Japan) and processed using ImageJ software.

### 2.11 Statistical analysis

Statistical analysis was performed with GraphPad Prism 9. All data were presented as mean ± Standard error of the mean (SEM) from at least three independent experiments. Differences between two groups were compared using a two-tailed Student’s t-test and Normal distribution was determined using D’Agostino and Pearson normality test. One-way ANOVA with Dunnett’s test for multiple comparisons was used to analyze the differences in more than two groups as appropriate. Survival curves for mice were calculated according to the Kaplan–Meier method; survival analysis was performed using the log-rank test. *, P < 0.05; **, P < 0.01; ***, P < 0.001; ****, P < 0.0001; ns, not significant.

## 3 Results

### 3.1 Neutrophils adhering to the EC layer form NETs in response to PMA, LPS, or LTA

**Figure 1:**
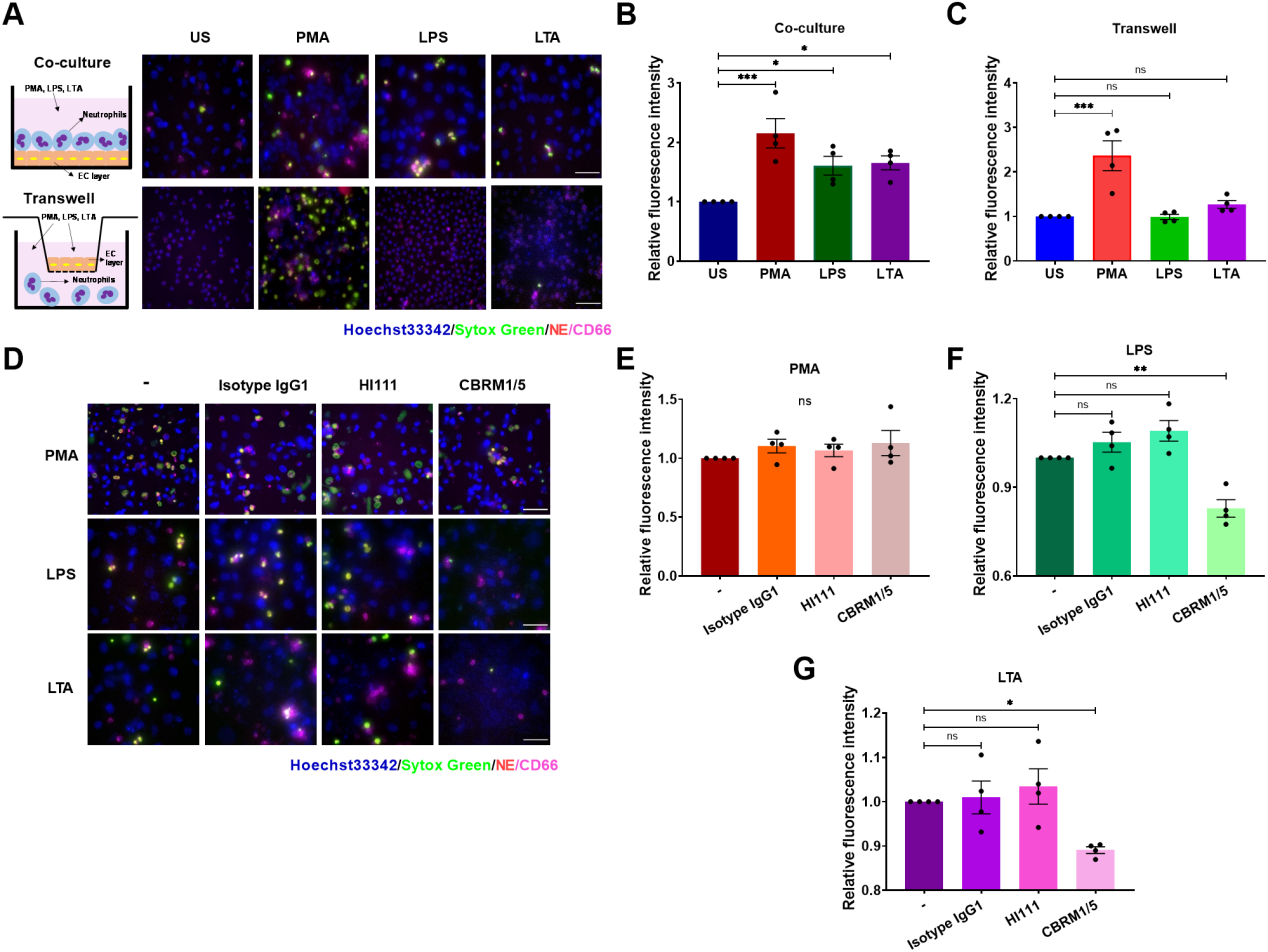
Stable adhesion is critical for EC-mediated NET formation in response to LPS or LTA. **(A)** NETs from unstimulated (US), and PMA- (100 ng/mL), LPS- (5 μg/mL), or LTA -(40 μg/mL) stimulated neutrophils were visualized by staining DNA with Sytox green (green) and NE (red), Nuclei were labeled by Hoechst 33342 (blue), and neutrophils were identified by an anti-CD66 antibody (magenta). Upper panel: Neutrophils were co-cultured with TNF-α pretreated ECs for 30 min before stimulation. Lower panel: Use of transwells to prevent direct contact between neutrophils and ECs. Scale bar, 50 µm. **(B, C)** Quantification of relative NET fluorescence intensity for co-culture **(B)** and transwell groups **(C)**. **(D)** Immunofluorescence images of NET formation post-LFA-1(HI111) or Mac-1(CBRM1/5). Scale bar, 50 μm. **(E–G)** Relative NET fluorescence intensity in response to PMA **(E)**, LPS **(F)**, or LTA **(G)**. Data represented as the mean ± SEM (n = 4). One-way ANOVA with Dunnett’s test for multiple comparisons was used to test statistical significance (*P < 0.05; **P < 0.01; ***P < 0.001; ns, not significant compared with the control group).

While the interplay between activated platelets and neutrophils in NET formation is well-documented, the role of endothelial cells in this process in sepsis remains unclear, although they are known to engage in adhesive interactions during thrombosis (43). To elucidate this, we co-cultured neutrophils with EC layers that had been pre-stimulated with tumor necrosis factor-α (TNF-α) and assessed NET production in response to PMA, LPS, or LTA. PMA, an agonist of protein kinase C, served as a positive control. LPS (activator of TLR4) and LTA (activator of TLR2) were selected to mimic the Gram-negative and Gram-positive bacterial infections, respectively. Neutrophils demonstrated a pronounced NET production when attached to the EC layer and stimulated with PMA, LPS, or LTA (**Figures 1A, B**). Notably, ECs did not release ETs upon PMA stimulation (**Supplementary Figure S1**). Interestingly, when neutrophils were separated from ECs in transwells, preventing direct contact, only PMA stimulation- neither LPS nor LTA-induced NET formation (**Figures 1A, C**). These results indicate stable adhesion of neutrophils on ECs is critical for NET formation in response to LPS or LTA.

The interactions between adhesion molecules, such as PSGL-1 binding to P-selectin and β_2_ integrins binding to ICAM-1, play critical roles in mediating the attachment of neutrophils to ECs (30). In addition, P-selectin and β_2_ integrins are implicated in NET formation (36, 41, 44). We explored the influence of PSGL-1 and β_2_ integrins on NET formation by using specific antibodies to block PSGL-1 (KPL-1), LFA-1 (HI111), or Mac-1 (CBRM1/5). Blocking PSGL-1, LFA-1, or Mac-1 did not impede NET release upon PMA stimulation as expected (**Figures 1D, E**, **Supplementary Figure S2**). However, Mac-1, but not LFA-1 or PSGL-1, was essential for NET release in response to LPS or LTA, as NETs were inhibited solely by the CBRM1/5 antibody (**Figures 1D, F, G**, **Supplementary Figure S2**). A significant decrease in cell viability and an increase in surface ICAM-1 expression were observed when ECs were co-incubated with NETs released by neutrophils stimulated with LPS. However, EC injury was reduced following co-incubation with NETs released by neutrophils when Mac-1 was blocked (**Supplementary Figure S3**). These data demonstrate that neutrophil adhesion to ECs triggers NET formation via Mac-1 in the presence of LPS or LTA stimuli.

### 3.2 NET formation of neutrophils on ICAM-1 is Mac-1 dependent in response to LPS or LTA

**Figure 2:**
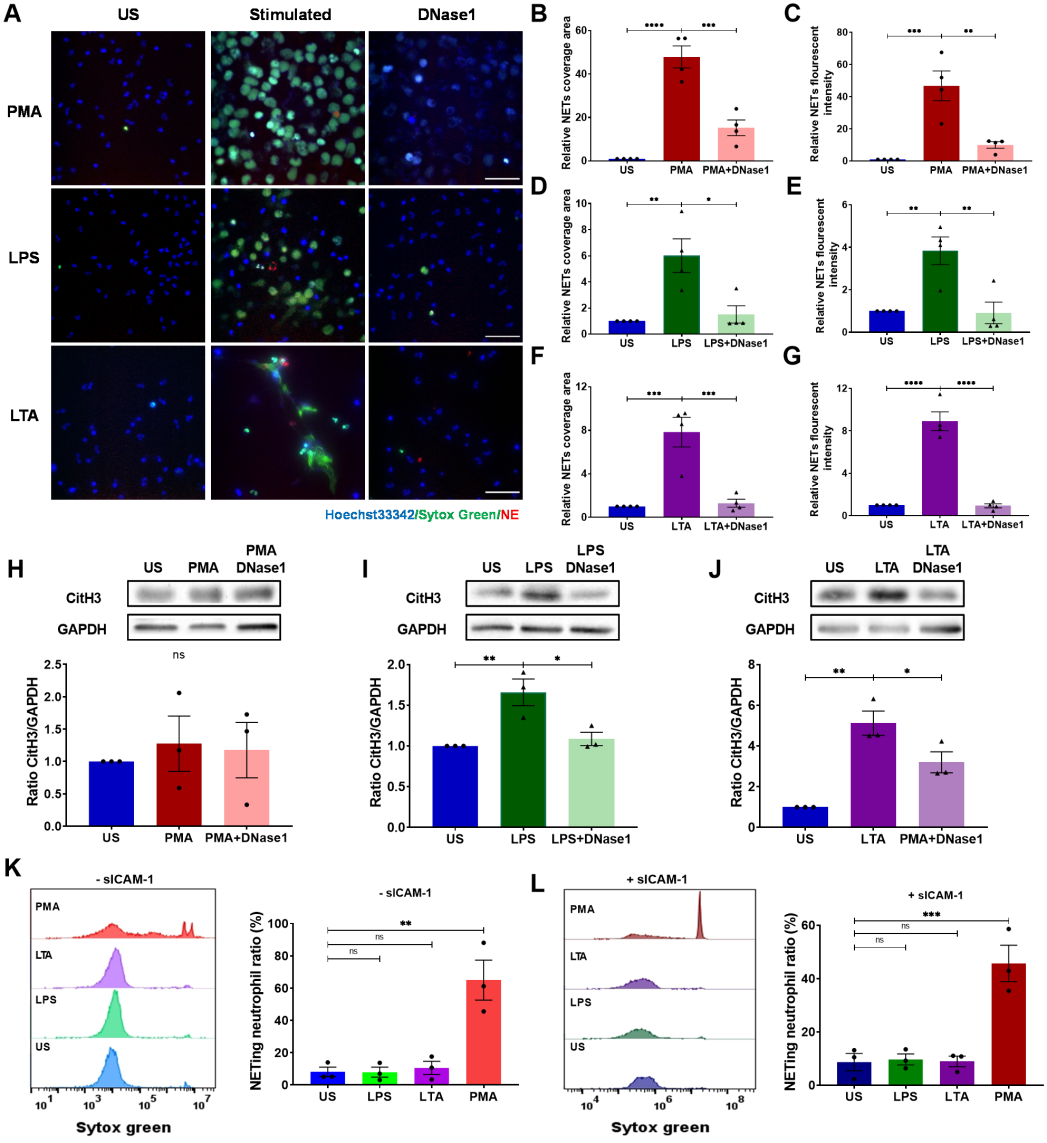
Neutrophils on ICAM-1-coated surface produce NETs upon LPS or LTA stimulation. Neutrophils attached to ICAM-1 produced NETs upon 4 h of stimulation with PMA, LPS, or LTA. Extracellular DNA was digested by DNase (I) **(A)** Visualization of NETs by staining DNA (Sytox green, green) and NE (red) in response to PMA, LPS, or LTA. Nuclei were labeled by Hoechst 33342 (blue). Scale bar, 50 μm. **(B, D, F)** Relative NET coverage area post- PMA **(B)**, LPS **(D)**, or LTA **(F)** stimulation with or without DNase I treatment. **(C, E, G)** Relative NET fluorescence intensity induced by PMA **(C)**, LPS **(E)**, or LTA **(G)** (n ). **(H–J)** Neutrophils were lysed and immunoblotted with an anti-CitH3 antibody. An anti-GAPDH antibody was used to indicate protein loading levels. Densitometry of CitH3 versus GAPDH was from three independent western blot experiments. **(K–L)** NETing neutrophil ratio was measured by flow cytometry from suspended neutrophils with or without soluble ICAM-1 (n = 3). In these experiments, unstimulated neutrophils (US) served as negative controls, and PMA-treated neutrophils as positive controls. Data represented as the mean ± SEM. One-way ANOVA with Dunnett’s test for multiple comparisons was used to test statistical significance (*P < 0.05; **P < 0.01; ***P < 0.001; ****P < 0.0001; ns, not significant compared with the control group).

To elucidate the mechanism of EC-mediated adhesion-dependent NET formation, we employed a simplified model by seeding neutrophils on surfaces pre-coated with ICAM-1. Unstimulated neutrophils failed to produce NETs and served as negative controls (**Supplementary Video S1**). PMA stimulation led to substantial NET production after 4 h stimulation (**Figures 2A–C**; **Supplementary Video S2**), while LPS (**Figures 2A, D, E**; **Supplementary Video S3**) or LTA (**Figures 2A, F, G**; **Supplementary Video S4**) induced lesser, yet notable, NET formation. Consistent with prior studies, adding DNase-I fragmented NET-DNA, releasing NE, and markedly reducing the area and fluorescence intensity of DNA (**Figures 2B–G**). CitH3 levels, as assessed by Western blot, showed results consistent with staining experiments, except in the PMA group, where the NET release was independent of CitH3 (**Figures 2H–J**). Interestingly, neutrophils in suspension formed NETs only in response to PMA, irrespective of soluble ICAM-1, while neither LPS nor LTA prompted NET formation (**Figures 2K, L**). These results indicate that neutrophil adhesion is crucial in ICAM-1-mediated NET formation induced by LPS or LTA.

**Figure 3:**
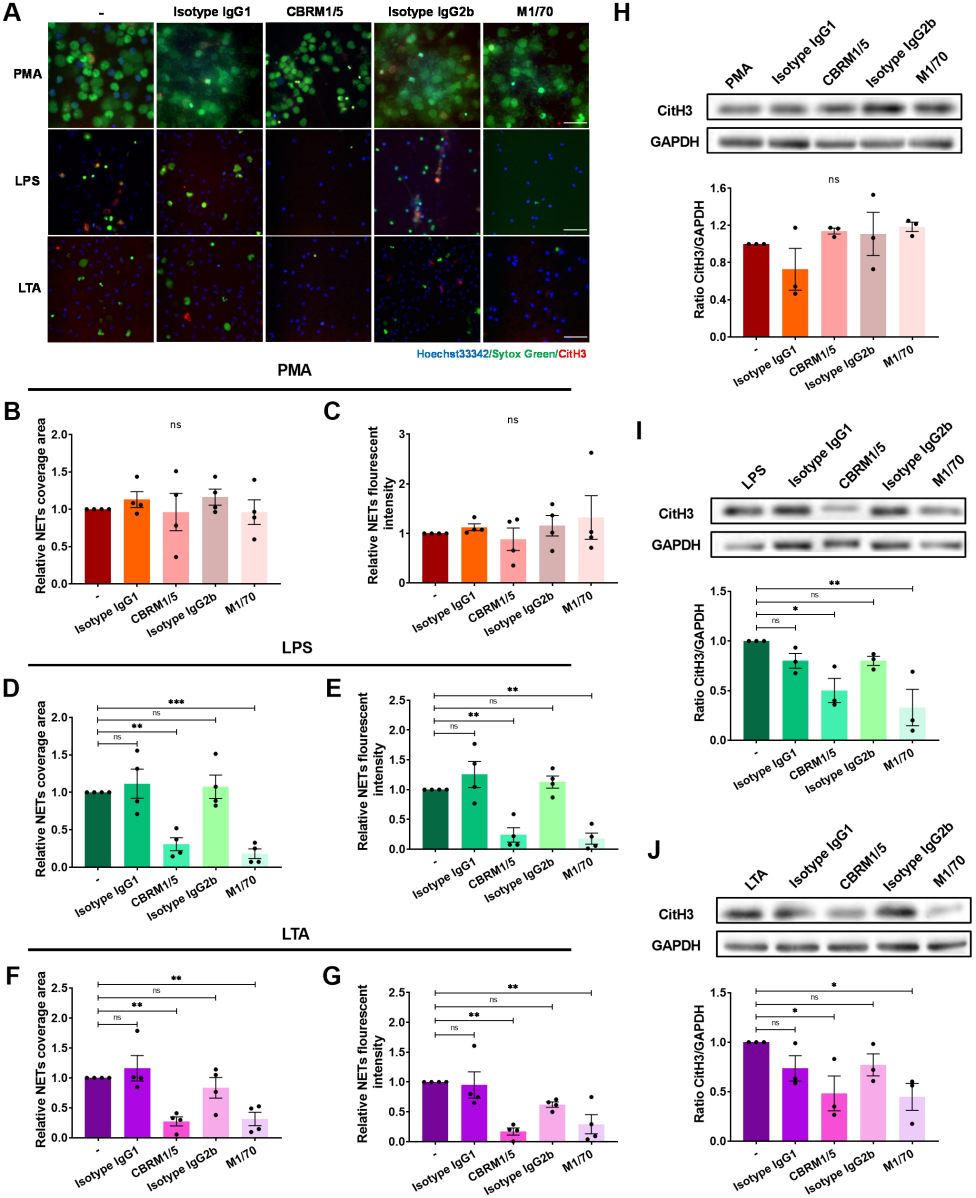
Impairment of NET formation by Mac-1 inhibition. Neutrophils without pre-treatment or pre-treated with the anti-Mac-1 antibodies CBRM1/5 or M1/70, or the isotype IgG, were stimulated by PMA, LPS, or LTA for 4 h at 37°C. **(A)** Visualization of NETs by staining DNA (Sytox green, green) and CitH3 (red), nuclei with Hoechst 33342 (blue). Scale bar, 50 μm. **(B, D, F)** Relative NET coverage area induced by PMA **(B)**, LPS **(D)**, or LTA **(F)** after blocking Mac-1. **(C, E, G)** Relative NET fluorescence intensity induced by PMA **(C)**, LPS **(E)**, or LTA **(G)**. Data represented as the mean ± SEM (n = 4 ). **(H–J)** Neutrophils were lysed and immunoblotted with an anti-CitH3 antibody. An anti-GAPDH antibody was used to indicate protein loading levels. Densitometry of CitH3 versus GAPDH (n = 3). One-way ANOVA with Dunnett’s test for multiple comparisons was used to test statistical significance (*P < 0.05; **P < 0.01; ***P < 0.001; ns, not significant compared with the control group).

To further assess the role of integrins on NET formation, neutrophils were pre-treated with anti-Mac-1 antibodies CBRM1/5, M1/70, or isotype control, and adhered on ICAM-1 layer followed by the stimulation of PMA, LPS, or LTA. The results demonstrated that Mac-1 was not necessary for PMA-induced NET formation (**Figures 3A–C**; **Supplementary Video S5**). However, employing CBRM1/5 or M1/70 antibodies significantly attenuated the DNA area and fluorescence intensity in response to LPS or LTA (**Figures 3A, D–G**; **Supplementary Video S6**, **7**). Similar results were observed in Western blot experiments measuring CitH3 levels (**Figures 3H–J**). Consistent with the results obtained from the EC layer experiments, LFA-1 inhibition via HI111 did not impact NET formation in response to PMA, LPS, or LTA (**Supplementary Figure S4**). Collectively, these results underscore Mac-1’s critical role in mediating NET formation in response to LPS or LTA.

### 3.3 Septic patient plasma induces NET formation via Mac-1 but not LFA-1

**Figure 4:**
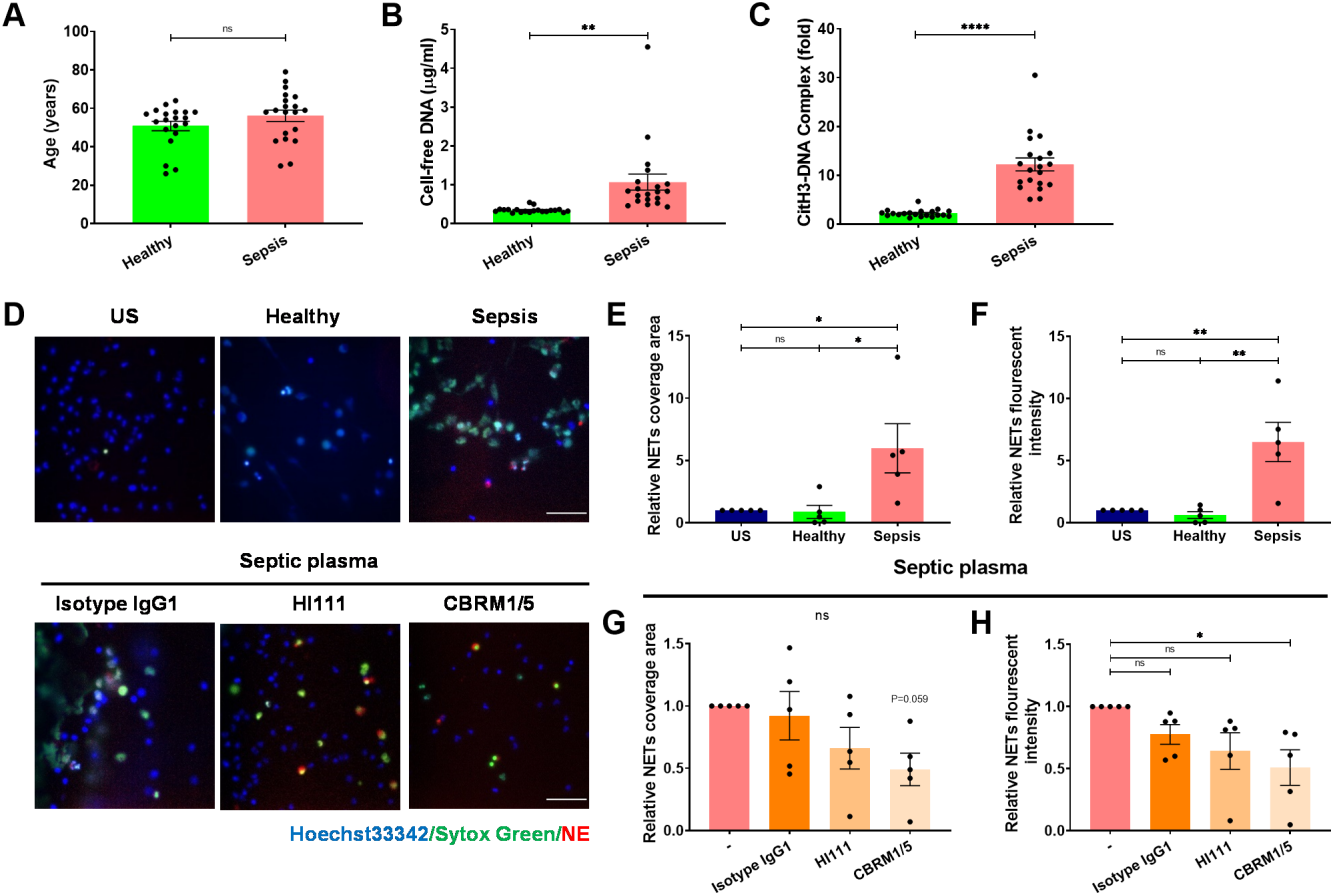
The septic plasma induces NET release via Mac-1 but not LFA-1. **(A)** Age distribution of healthy individuals and septic patients. **(B, C)** Comparison of plasma NET markers cell-free DNA **(B)** and CitH3-DNA complex **(C)** between healthy individuals (n = 20) and septic patients (n = 20). **(D)** Upper panel: Representative microphotographs showing NET release from healthy neutrophils treated with plasma from healthy controls or septic patients. Lower panel: Representative images of neutrophils pre-treated with HI111, CBRM1/5, or the isotype control and stimulated with septic plasma for 4 h at 37°C. NETs were stained for DNA (Sytox green, green) and NE (red), nuclei with Hoechst 33342 (blue). Scale bar, 50 μm. **(E, F)** Quantification of relative NET coverage area **(E)** and fluorescence intensity **(F)** for healthy neutrophils treated with or without healthy or septic plasma. **(G, H)** Quantification of relative NET coverage area **(G)** and fluorescence intensity **(H)** of neutrophils pre-treated with the antibodies HI111, CBRM1/5, or isotype IgG, induced by septic plasma (n = 5). Data represented as the mean ± SEM. Two-tailed Student’s t-test and One-way ANOVA with Dunnett’s test for multiple comparisons were used to test statistical significance, respectively (*P < 0.05; **P < 0.01; ****P < 0.0001; ns, not significant compared with the control group).

To investigate the potential of Mac-1 inhibition in mitigating NET formation during sepsis, plasma from 20 adult sepsis patients was examined. Patient demographics were shown in **Table 1**, including an average age of 56.1 ± 3.0 years with even gender distribution (50% male), while the healthy control group averaged 50.8 ± 2.5 years with 50% male composition (**Figure 4A**). NET markers in circulation, such as cell-free DNA and CitH3-DNA complex, were significantly elevated in patient samples compared to those from healthy individuals (**Figures 4B, C**). Compared to those treated with healthy plasma, neutrophils exposed to septic patient plasma exhibited a substantial increase in NET production, as demonstrated by the relative NET coverage area and fluorescent intensity (**Figures 4D–F**). Notably, Mac-1 inhibition with CBRM1/5 significantly reduced NET formation induced by septic plasma (NET coverage reduced to 0.49-fold, and NET fluorescence intensity reduced to 0.5-fold) (**Figures 4D, G, H**). Inhibition of LFA-1 by HI111, however, did not affect NET formation (**Figures 4D, G, H**). These results indicate that septic patient plasma induces NET formation via Mac-1, rather than LFA-1.

### 3.4 PAD4, but not ROS, contributes to the NET formation

**Figure 5:**
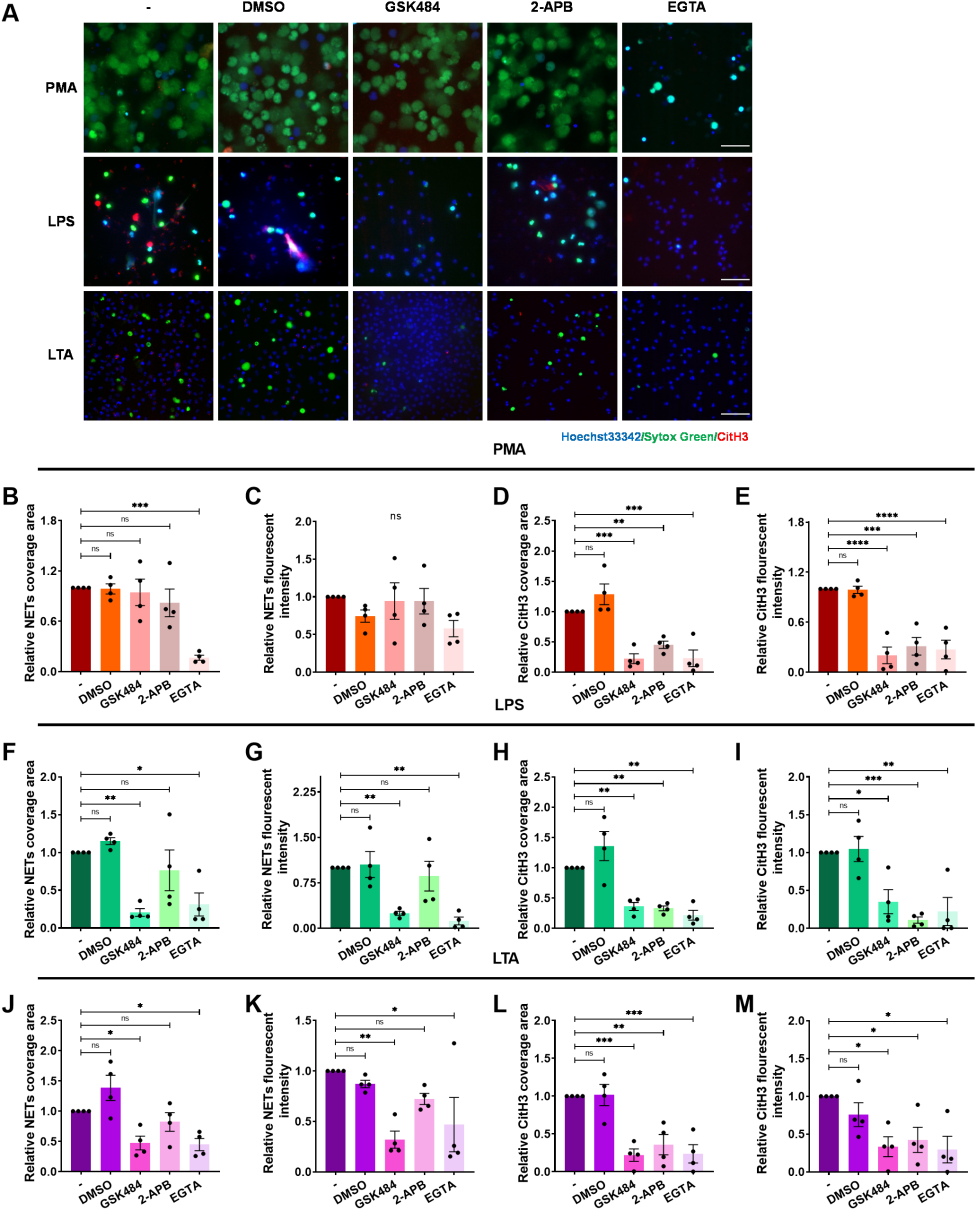
Regulation of the of histone H3 citrullination and NET formation by extracellular calcium influx. Neutrophils, pre-incubated with the PAD4 inhibitor GSK484, IP3 inhibitor 2-APB, calcium chelator EGTA, or vehicle control DMSO, were stimulated with PMA, LPS, or LTA for 4 h at 37°C. **(A)** Visualization of NETs by staining DNA (Sytox green, green) and CitH3 (red), nuclei with Hoechst 33342 (blue). Scale bar, 50 μm. **(B–E)** Quantification of NETs **(B, C)** and CitH3 **(D, E)** in response to PMA. **(F–I)** Quantification of NETs **(F, G)** and CitH3 **(H, I)** in response to LPS. **(J–M)** Quantification of NETs **(J, K)** and CitH3 **(L, M)** in response to LTA. Data represented as the mean ± SEM from (n = 4). One-way ANOVA with Dunnett’s test for multiple comparisons was used to test statistical significance (*P < 0.05; **P < 0.01; ***P < 0.001; ****P < 0.0001; ns, not significant compared with the control group).

Arginine deamination has been implicated in NET formation, as inhibition or genetic absence of PAD4 impedes this process (45). To investigate the role of PAD4 in ICAM-1-Mac-1-mediated NET release, neutrophils were treated with the selective PAD4 inhibitor GSK484 prior to PMA, LPS, or LTA exposure. The extracellular DNA and histone H3 citrullination were detected by fluorescence microscopy (**Figure 5A**). PAD4 inhibition did not affect PMA-induced NETs, despite reduced CitH3 levels (**Figures 5B–E**). However, GSK484 significantly curtailed NET-DNA and CitH3 signals following LPS (**Figures 5F–I**) or LTA (**Figures 5J–M**) stimulation, indicating PAD4’s necessity for NET release under these conditions. Furthermore, Mac-1 inhibition reduced histone H3 citrullination (**Supplementary Figure S5**), suggesting Mac-1’s regulatory role over PAD4-mediated citrullination in NET formation, in contrast to LFA-1, which did not show such an effect (**Supplementary Figure S6**).

Given PAD4’s calcium dependency (46), the influence of Ca^2+^ on Mac-1-mediated NET formation was also investigated. 2-APB was employed to inhibit Ca^2+^ release from the endoplasmic reticulum and DMSO as the vehicle control. Extracellular calcium was chelated with EGTA to prevent Ca^2+^ influx. We observed that 2-APB did not significantly diminish NET formation induced by PMA-, LPS-, or LTA (**Figures 5B, C, F, G, J, K**), but did reduce CitH3 (**Figures 5D, E, H, I, L, M**). However, EGTA treatment notably inhibited both DNA release and CitH3 in response to PMA, LPS, or LTA (**Figures 5B–M**), underscoring the importance of extracellular Ca^2+^ influx in NET formation under these conditions.

**Figure 6:**
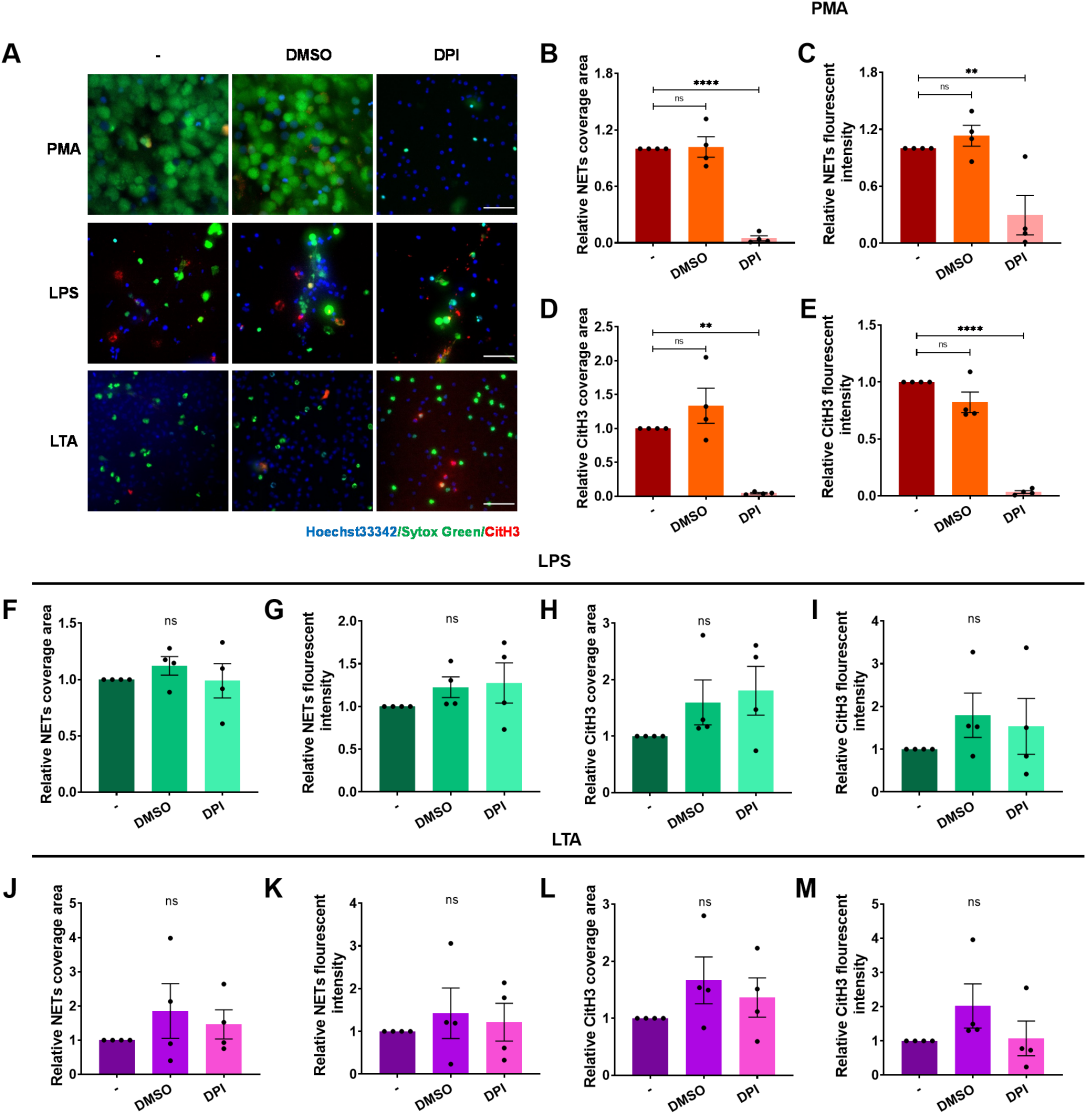
ROS is dispensable for ICAM-1-mediated NET formation upon LPS or LTA stimulation. Neutrophils, pre-incubated with the inhibitors DPI, or vehicle control DMSO, were stimulated by PMA, LPS, or LTA for 4 h at 37°C. **(A)** Visualization of NETs by staining DNA (Sytox green, green) and CitH3 (red), nuclei with Hoechst 33342 (blue). Scale bar, 50 μm. **(B–E)** Quantification of NETs **(B, C)** and CitH3 **(D, E)** in response to PMA after ROS inhibition. **(F–I)** Quantification of NETs **(F, G)** and CitH3 **(H, I)** in response to LPS after ROS inhibition. **(J–M)** Quantification of NETs **(J, K)** and CitH3 **(L, M)** in response to LTA after ROS inhibition. Data represented as the mean ± SEM (n = 4 ). One-way ANOVA with Dunnett’s test for multiple comparisons was used to test statistical significance (**P < 0.01; ****P < 0.0001; ns, not significant compared with the control group).

Reactive oxygen species (ROS) are also regarded to be critical for NET formation induced by various stimuli (47). However, some evidence suggests ROS-independent pathways exist (48). We assessed ROS involvement in ICAM-1-Mac-1-mediated NET formation using DPI to halt NOX2-dependent ROS generation. DPI abolished NET release in response to PMA stimulation (**Figures 6A–E**), in line with previous studies (47). However, it failed to suppress NET formation induced by LPS or LTA (**Figures 6A, F–M**). Further investigations into intracellular ROS production confirmed that unlike PMA, LPS or LTA did not induce significant ROS production in neutrophils (**Supplementary Figure S7**). These findings demonstrate that NOX2-mediated ROS production is not essential for ICAM-1-Mac-1-driven NET formation in response to LPS or LTA.

### 3.5 Blocking Mac-1 impaired NET formation and ameliorated lung injury *in vivo*

**Figure 7:**
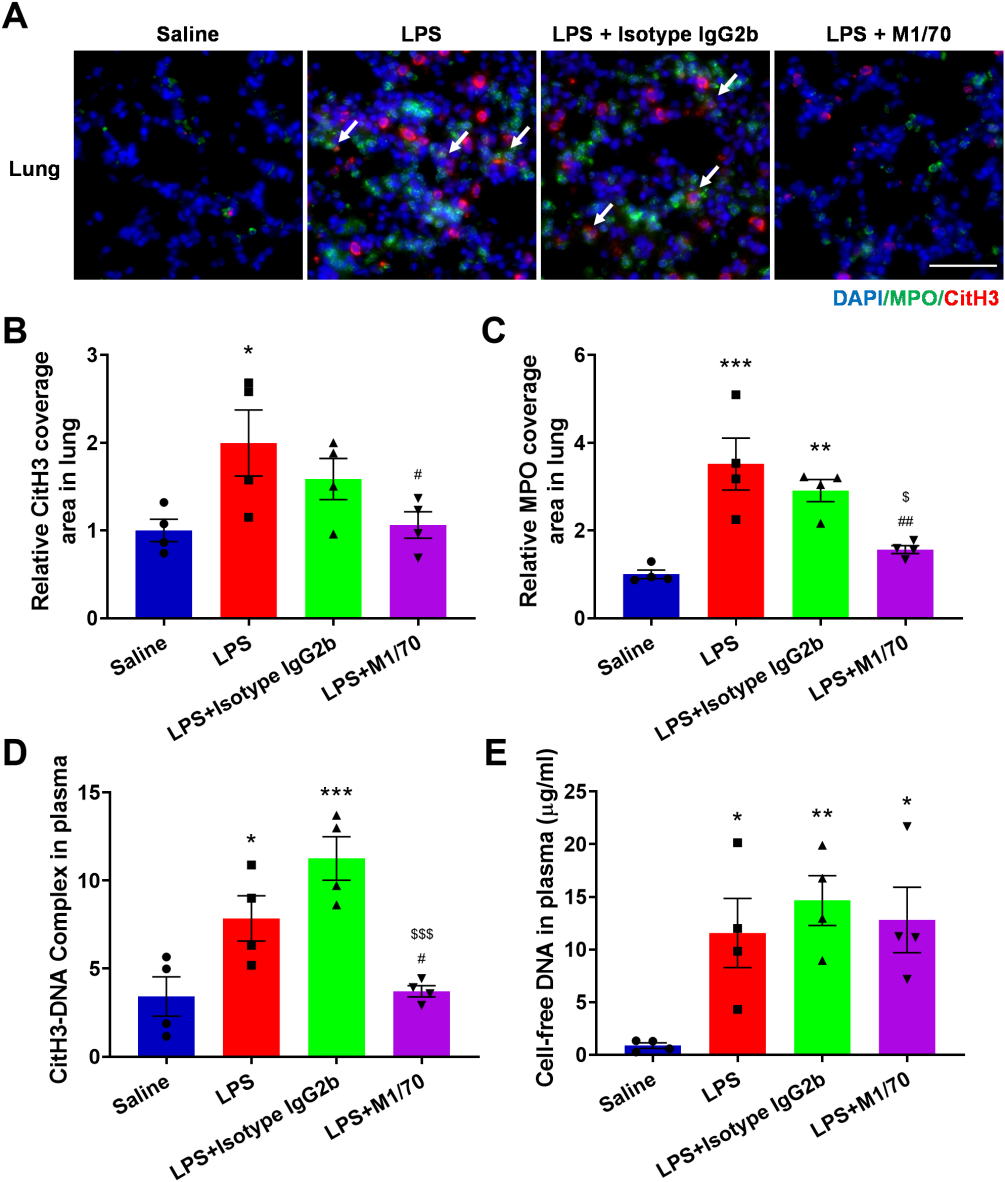
Impact of Mac-1 inhibition on NET formation. **(A)** Representative images of NETs stained with CitH3 (red) and MPO (green) in the lung tissues, nuclei with DAPI (blue). Arrows highlight co-location of CitH3 and MPO. Scale bar, 50 μm. **(B, C)** Quantification of relative CitH3 **(B)** and MPO **(C)** coverage area in the lungs. **(D, E)** Plasma levels of cell-free DNA **(D)** and CitH3-DNA complex **(E)** from control or therapeutic mice measured using ELISA 8 h post-LPS injection. Data represented as the mean ± SEM (n = 4 ). One-way ANOVA with Dunnett’s test for multiple comparisons was used to test statistical significance (*p < 0.05, **p < 0.01, ***p < 0.001 vs. Saline, ^#^p < 0.05, ^##^p < 0.01, vs. LPS, ^$^p < 0.05, ^$$$^p < 0.001 vs. LPS + Isotype IgG 2b, ns, not significant).

To further access the role of Mac-1 in NET release and the physiopathology of sepsis *in vivo*, male C57Bl/6J mice were induced with sepsis via intraperitoneal (i.p.) LPS injection. The Mac-1 inhibitory antibody M1/70 was intravenously introduced 30 min prior to the LPS challenge. The administration of these reagents did not affect neutrophil and monocyte counts in mice (**Supplementary Figure S8**). Immunofluorescence staining of CitH3 and MPO was performed to detect NETs in lung sections (**Figure 7A**). NETs were elevated in lungs post-LPS administration compared to the saline control group and notably reduced in the M1/70 pre-treated group, contrasting with isotype IgG (**Figures 7B, C**). Plasma NET makers, including cell-free DNA (**Figure 7D**) and CitH3-DNA complex (**Figure 7E**), were elevated in LPS-challenged mice. Mac-1 inhibition abrogated the CitH3-DNA complex in plasma, although no significant change in cell-free DNA, possibly because NETs were not the only source of cell-free DNA in plasma (**Figures 7D, E**). M1/70 administration alleviated LPS-induced endothelial injury in lung tissue, which is known to be susceptible to NETs (**Supplementary Figure S9**). These results suggest that blocking Mac-1 reduces NET release and endothelial injury.

**Figure 8:**
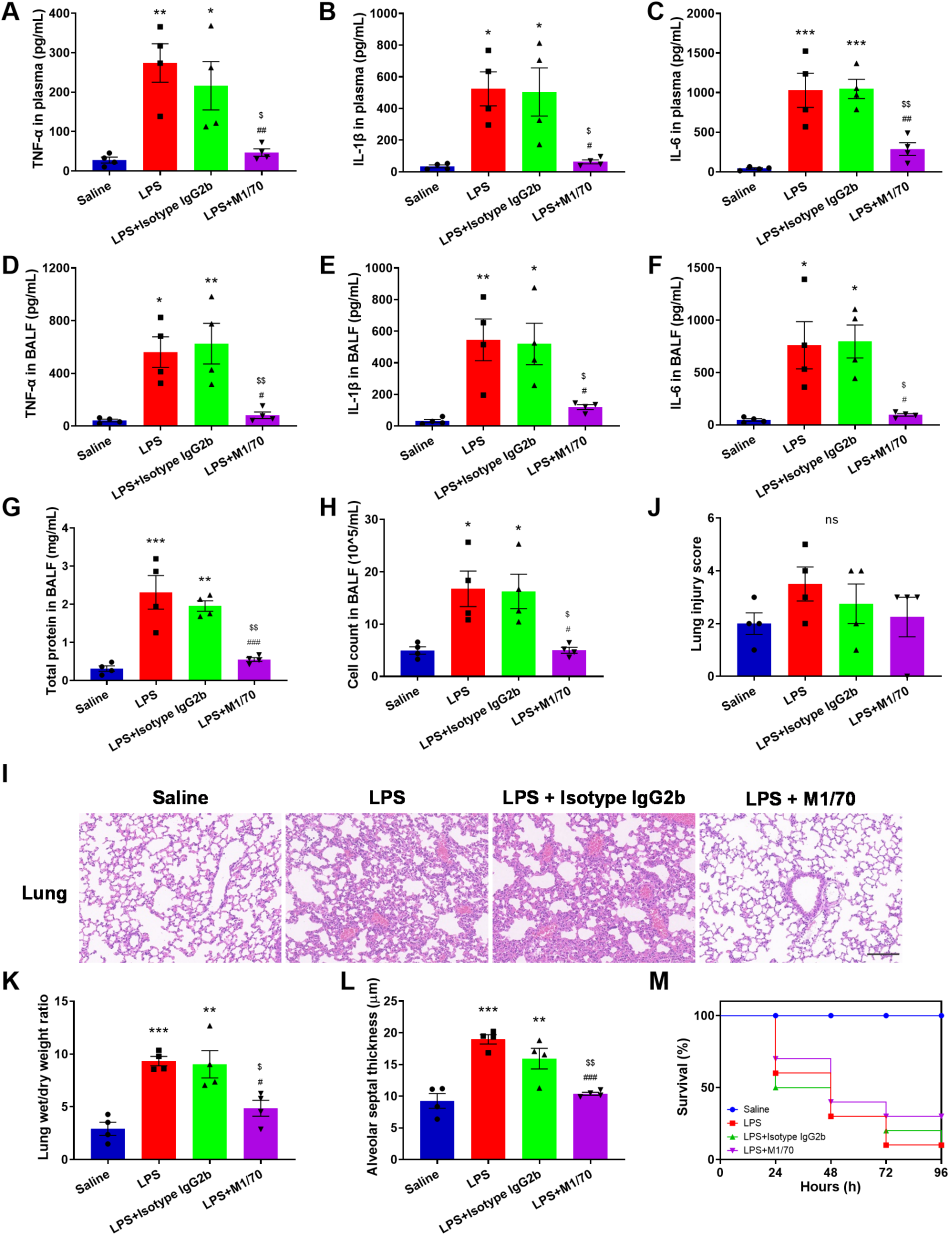
Blocking Mac-1 alleviates lung injury in LPS mice. **(A–F)** Quantification of indicated cytokines (TNF-α, IL-1β, and IL-6) in the plasma **(A–C)** and BALF **(D–F)**. **(G, H)** Quantification of total protein content **(G)** and cell counts **(H)** in the BALF. **(I)** Lung histopathology was assessed by H&E staining. Scale bar, 100 μm. **(J–L)** Lung injury scores **(J)**, lung wet/dry weight ratio **(K)**, and alveolar septal thickness **(L)** evaluations. Data represented as the mean ± SEM (n = 4 ). **(M)** Kaplan–Meier survival curves for M1/70-, Isotype IgG2b-treated, and LPS control mice. Saline was set as a negative control (n = 10). Survival analysis was performed using the log-rank test. One-way ANOVA with Dunnett’s test for multiple comparisons was used to test statistical significance (*p < 0.05, **p < 0.01, ***p < 0.001 vs. Saline, ^#^p < 0.05, ^##^p < 0.01, ^###^p < 0.001 vs. LPS, ^$^p < 0.05, ^$$^p < 0.01 vs. LPS + Isotype IgG 2b vs. LPS + Isotype IgG 2b, ns, not significant).

Analysis of plasma and BALF revealed markedly increased levels of proinflammatory cytokines, TNF-α, IL-1β, and IL-6, in LPS-challenged mice (**Figures 8A–F**). Blocking Mac-1 significantly reduced the levels of these proinflammatory cytokines in both plasma and BALF (**Figures 8A–F**). Additionally, a remarkable increase in total protein content and cell counts in the BALF was detected in response to LPS, which was inhibited by M1/70 pretreatment (**Figures 8G, H**). Given the association between NETs and organ injury and sepsis severity (49, 50), we investigated the role of Mac-1 in septic lung injury. Histopathological assessment revealed LPS-induced alveolar wall thickening and alveolar interval enlargement after 8 h (**Figure 8I**). Although the decrease in lung injury score was not significant (**Figure 8J**), M1/70 administration markedly lessened the lung wet/dry weight ratio (**Figure 8K**) and alveolar septum thickness (**Figure 8L**). In the subsequent Kaplan–Meier survival analysis, we found Mac-1 blockade improved the survival rate (30% vs. 10% in LPS controls), although the difference was not statistically significant (**Figure 8M**). Collectively, these findings suggest that Mac-1 inhibition not only reduces NET production but also attenuates systemic inflammation and lung injury during sepsis, improving survival.

## 4 Discussion

In this study, we have demonstrated the crucial role of Mac-1 in the formation of NETs by neutrophils stably adhering to ECs or an ICAM-1 layer under exposure to LPS, LTA, or septic plasma. This Mac-1-mediated NET formation is dependent on PAD4-induced histone citrullination and the influx of extracellular calcium, rather than ROS. Inhibition of Mac-1 disrupted NET formation induced by LPS, LTA, or septic plasma *in vitro*, and ameliorated lung injury *in vivo* in a model of LPS-induced sepsis. These findings position Mac-1 as a potential therapeutic target for improving sepsis treatment outcomes.

Platelet, neutrophil, and EC activation coordinate the progression of thrombo-inflammatory during infection (51). Most studies have concentrated on activated platelet-mediated NET formation. For instance, platelet TLR4 has been shown to promote neutrophil activation and NET formation in endotoxemia and Gram-negative bacterial sepsis (16). In contrast, research on EC-mediated NET formation is limited. It has been reported that activated ECs can indirectly induce NET formation through the release of IL-8 or miR-505 (23, 24). Given that neutrophils interact directly with ECs when recruited from blood to infected tissue, it is essential to determine whether this interaction can induce NET formation under infectious conditions. Our research found that neutrophils co-cultured with ECs formed NETs upon LPS or LTA stimulation (**Figure 1B**), and the NET formation still occurred when the endothelial layer was replaced with an ICAM-1 layer (**Figures 2A–J**). It is worth noting that the NET release was abolished after preventing the direct contact of neutrophils with ECs (**Figure 1C**). Furthermore, neutrophils in suspension were unable to form NETs in the presence of soluble ICAM-1 in response to LPS or LTA (**Figures 2K, L**). These results strongly demonstrate that stable adhesion of neutrophils to ECs is critical for NET formation during infection.

Integrins mediate the stable adhesion of neutrophils to ECs by binding to up-regulated ICAM-1 on ECs during sepsis (52). Integrins also are involved in NET formation in various scenarios (35, 53). β_2_ integrins have been identified as a master switch for NET induction (39). In mice and human septicemia, the release of intravascular NETs is dependent on LFA-1-mediated platelet-neutrophil interactions (41, 54). In ALI, the release of NETs depends on signaling mediated by Mac-1 and G protein-coupled receptors (40). PMA-induced NET formation is independent of Mac-1, while activated Mac-1 is necessary for antiphospholipid syndrome IgG-mediated NET release (55). In contrast to LFA-1, our data demonstrated that Mac-1 is vital for NET formation when neutrophils stably adhere to ECs or an ICAM-1 layer and are stimulated by LPS or LTA (**Figures 1**, **3**). To further explore the relevance of this Mac-1-mediated NET formation during sepsis, septic plasma was used to stimulate normal neutrophils on an ICAM-1 layer, resulting in significant NETs, which were markedly reduced by blocking Mac-1. These findings demonstrate the critical role of Mac-1 in NET formation during sepsis (**Figure 4**). Although our data emphasize the importance of Mac-1–ICAM-1 binding in NET formation, we cannot exclude the possibility that EC-derived factors may synergistically promote NET formation with Mac-1 signaling. Future studies employing proteomics could provide a more comprehensive understanding.

The PAD4-dependent (45, 56) and ROS-dependent (57, 58) signaling pathways are the main signaling pathways for NET formation, but they are not mutually exclusive. Aspergillus fumigatus-induced NET formation requires both Mac-1 and ROS, independent of histone citrullination (36). NET formation against *C. albicans* hyphae depends on β-glucan recognition by Mac-1, requiring fibronectin and extracellular regulated kinase but not ROS (38). The NET formation induced by physiological agonists is ROS-independent, and prevented by selective inhibition of PAD4 (48). It has been suggested that NET formation is largely independent of endogenous ROS, but PAD4 involvement is crucial (56). Our data align with previous findings suggesting that NET formation in response to LPS or LTA relies on PAD4, with endogenous ROS playing a non-essential role (**Figures 5**, **6**).

**Figure 9:**
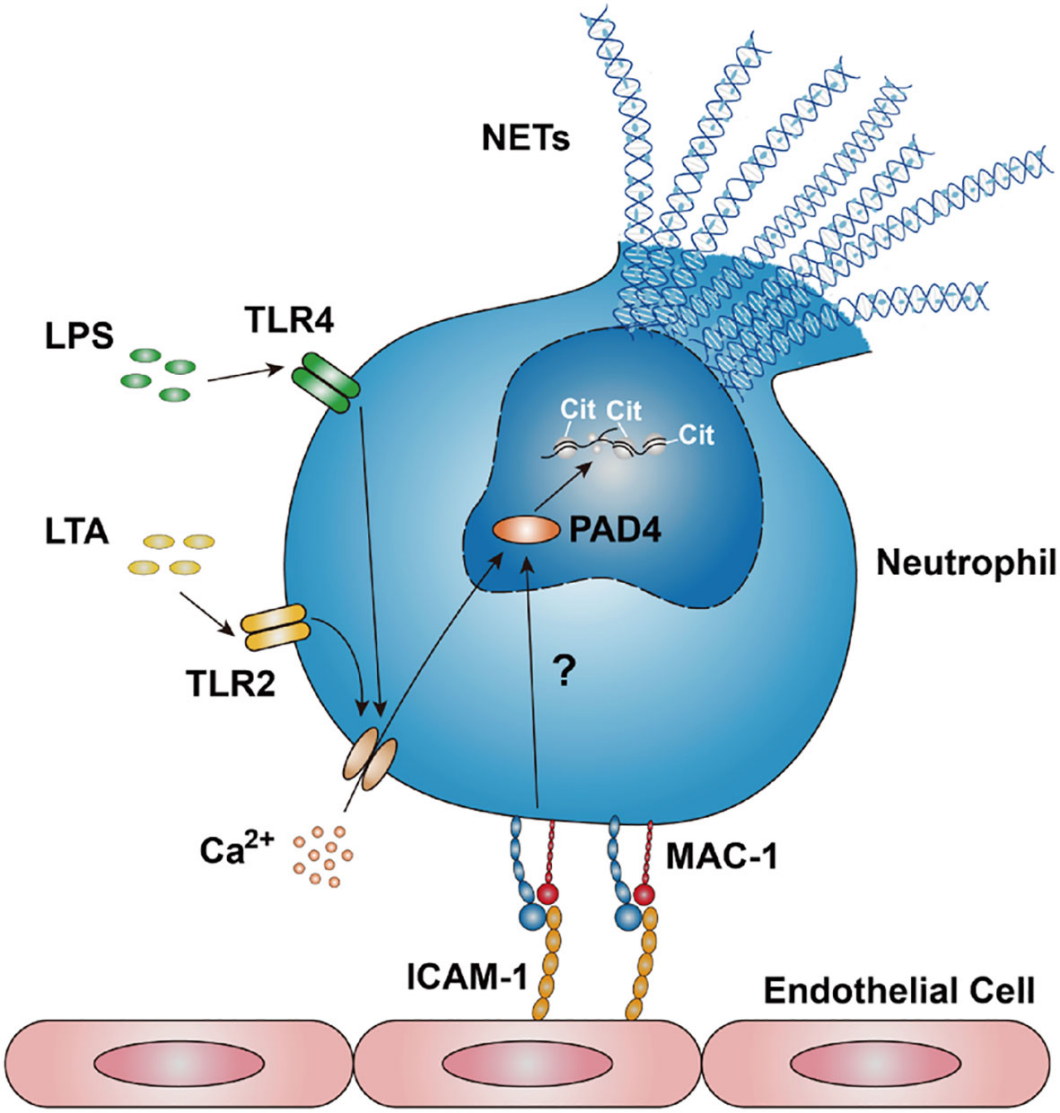
Schematic of ICAM-1-Mac-1 mediated NET formation mechanism in response to LPS or LTA. Neutrophils stimulated by LPS or LTA induces an influx of extracellular Ca^2+^, resulting in elevated intracellular Ca^2+^ levels. The interaction between endothelial ICAM-1 and neutrophil Mac-1 triggers an undefined signaling pathway. This pathway, along with Ca^2+^, synergistically regulates the citrullination of histone H3, thereby promoting NET formation.

Our research has demonstrated that LPS or LTA induces a gradual increase in intracellular Ca^2+^ concentration (**Supplementary Figure S10**), consistent with previous studies (59). Recently, we have shown that the engagement of β_2_ integrins with ICAM-1 or glycoprotein Ibα (GPIbα) induced the influx of extracellular Ca^2+^ (60). This study illustrates that NET formation in response to LPS or LTA depends on the influx of extracellular Ca^2+,^ rather than the release of stored intracellular stored Ca^2+^ (**Figure 5**). Notably, human PAD4 has five Ca^2+^ binding sites and requires binding with 5-10 mM Ca^2+^ to achieve the maximum activation effect. In the physiological environment, the intracellular Ca^2+^ concentration ranges from 10 nM to 100 μM, which is far less than that required for complete activation of PAD4. It has been suggested that Ca^2+^ in blood and plasma can synergistically activate PAD4 with other substances (61). We suspect that Mac-1 and Ca^2+^ might synergistically regulate PAD4 activation, inducing NET formation. This is supported by the evidence (1): blocking Mac-1 or chelating Ca^2+^ impedes PAD4-mediated citrullination of histone H3 (**Figure 5**, **Supplementary Figure S5**) (2); blocking Mac-1 effectively reduced NET formation and CitH3 levels without affecting intracellular Ca^2+^ levels (**Figure 3**, **Supplementary Figure S10**), suggesting a Ca^2+^-independent regulatory mechanism by Mac-1. We propose that LPS or LTA stimulation induce the influx of extracellular Ca^2+^, resulting in elevated intracellular Ca^2+^ levels in neutrophils; ICAM-1-Mac-1 interaction initiates an undefined Ca^2+^-independent pathway, which with Ca^2+^ synergistically mediate the citrullination of histone H3 by PAD4; and NETs were released eventually (**Figure 9**).

Mac-1 is critical in neutrophil adhesion, migration, and phagocytosis, and is implicated in various diseases, making it an attractive therapeutic target (62). Antibodies and small molecules targeting the interaction between Mac-1 and GPIbα have shown promise in inhibiting thrombosis (63). A monoclonal antibody, anti-M7, specifically blocks the interaction of Mac-1 with CD40L resulting in reduced leukocyte recruitment and cytokine secretion, improving sepsis outcomes by preventing excessive inflammation and enhancing bacterial clearance (64). A Designed-Ankyrin-Repeat-Protein, named F7, specifically targeting activated Mac-1, demonstrates therapeutic anti-inflammatory effects in mouse models of sepsis, myocarditis, and ischemia/reperfusion injury (65). NETs are implicated in the progression of many diseases. Increased cell-free DNA levels in septic patient plasma have been linked to increased mortality (66). Degradation of NETs by DNase infusion reduces intravascular coagulation, improves microvascular perfusion, and mitigates organ damage during sepsis (1). DNase and PAD4 treatment reduce NETs, improving lung injury and survival in a murine model of pneumonia (67). In this study, Mac-1 blockade was shown to reduce NET formation, lower inflammatory cytokine levels, mitigate endothelial damage, alleviate lung injury, and improve survival in a mouse model of LPS-induced sepsis (**Figures 7**, **8**). The trend toward improved survival, albeit statistically underpowered, supports that Mac-1 is a potential therapeutic target. Genetic approaches, such as Mac-1 knockout or knockdown, should applied to further confirm these findings in the future. Of note, it has been reported that Mac-1 deficiency worsens sepsis outcomes (68). We suggest that targeting a specific subset of Mac-1, such as activated Mac-1, or Mac-1-ligand interaction might be a potential strategy to improve the therapeutic outcomes.

Collectively, our study is the first, to our knowledge, to demonstrate that direct contact between ECs and neutrophils mediates NET formation via ICAM-1-Mac-1 interaction in response to LPS or LTA. This interaction initiates a Ca^2+^-independent signaling pathway, which, along with the influx of extracellular Ca^2+^, regulates PAD4 citrullinating histone H3 and subsequent NET release. Most importantly, we have shown that Mac-1 blockade reduces NET formation and ameliorates lung injury *in vivo*, suggesting Mac-1 is a potential target for improving sepsis treatment.

## Data Availability

This study includes no data deposited in external repositories. The data that support the findings of this study are available from the corresponding author upon reasonable request. Requests to access the datasets should be directed to JL, linjiangguo@gdph.org.cn.
