# Supplementary material for: Mac-1 blockade impedes adhesion-dependent neutrophil extracellular trap formation and ameliorates lung injury in LPS-induced sepsis
Source: Front Immunol. 2025 Mar 28;16:1548913. doi: 10.3389/fimmu.2025.1548913 (PMC11985419; doi:10.3389/fimmu.2025.1548913)
Supplement: Supplementary file 1 [file DataSheet1.docx]

Supplementary Material

# Supplementary Methods

Intracellular ROS measurements

Neutrophil intracellular ROS levels were measured by the microporous plate at 485nm (excitation) and 530 nm (emission) of 5μM CM-H2DCFDA. Under dark conditions, Neutrophils were pretreated with CM-H2DCFDA under dark conditions. Subsequently, PMA (100 μg/mL), LPS (100 μg/mL), or LTA (4 μg/mL) was added to stimulate neutrophils, and an equal volume of cell medium was added to the Blank control group. The 96-well plate was immediately placed on the Perkin Elmer multifunctional enzyme label and recorded every 10 minutes for 120 min.

HUVECs damaged by NETs

Neutrophils were stimulated with 100 ng/mL LPS for 4h, and then the supernatant was removed and the cells were washed with cold PBS, the NETs adhered at the bottom were collected for use. HUVECs were treated with conditional medium (LPS-stimulated neutrophil media) for 24 h, and the morphology and other related markers were evaluated. RPMI-1640 containing 10% FBS medium as Blank control. A cell counting Kit-8 (GOONIE, Guangzhou, China) was used to test relative cell viability according to the manufacturer's instructions, and the expression levels of ICAM-1 were detected by flow cytometry. Equal-volume of NET media derived from CBRM1/5 pretreated neutrophils were used to investigate the effect of Mac-1 inhibition on endothelial injury.

Intracellular Ca^2+^ signal measurements

Intracellular Ca^2+^ signals from neutrophils exposed to PMA (100 μg/mL), LPS (100 μg/mL), or LTA (4 μg/mL) were obtained by the microporous plate. Neutrophils were preloaded with the Calbryte-520 AM dye and Hochest 33342 for 30 min in a 96-well plate. After the cells were stimulated, fluorescence was monitored immediately on a fluorescence microplate reader with excitation/emission wavelength at 490 nm/525 nm of Calbryte-520 dye at 37°C for 10 min.

Live-cell video for NETs formation

Neutrophils (4×10^5^ cells/well) were seeded on the glass surface coated with ICAM-1 (100 μg/mL) and incubated for 30 min. After removing nonadherent neutrophils, PMA (100 ng/mL), LPS (100 ng/mL), or LTA (4 μg/mL) were added to stimulate adherent neutrophils. Hoechst 33342 and Sytox green were used to label nuclei and extracellular DNA respectively. Neutrophils were recorded by a 20× fluorescence microscope (Ti2-U, Nikon, Tokyo, Japan) at 5 min intervals for 4 h.

# Supplementary Figures

**
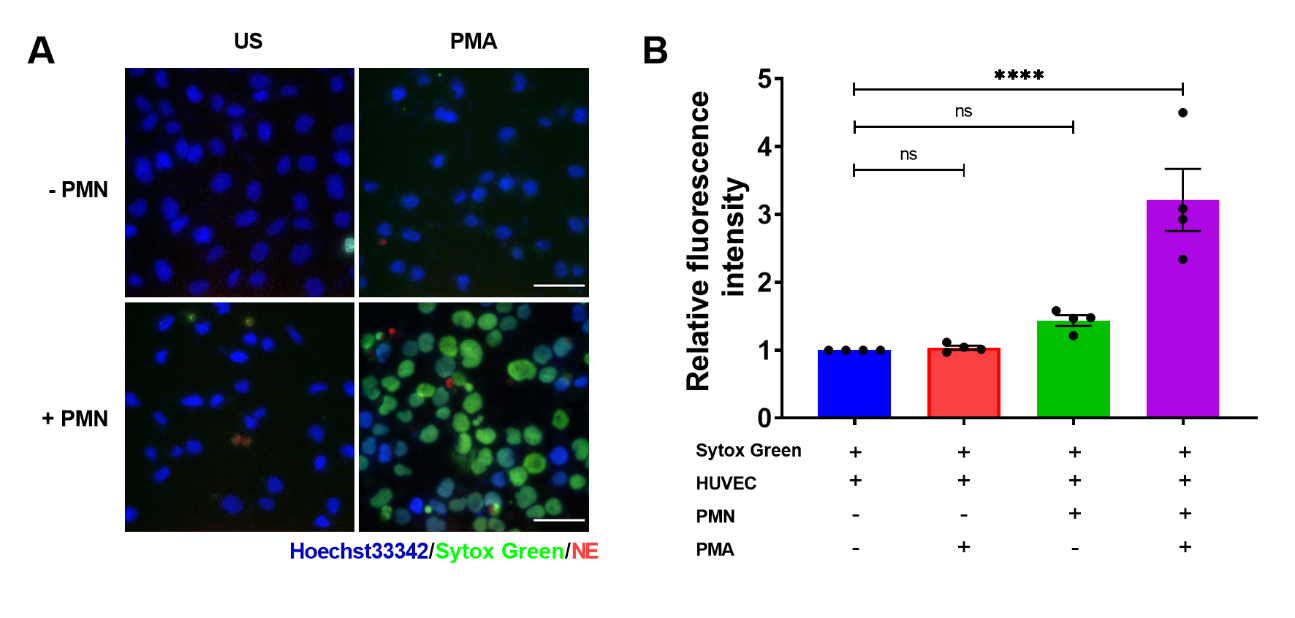
**

**Fig S1.** **HUVECs did not release ETs in response to PMA stimulation.** HUVECs were pretreated with TNF-α for 24 h and then incubated with neutrophils for 30 min before PMA stimulation. (A) ETs from US- (unstimulated) or PMA- (100 ng/mL) stimulated cells were visualized by staining DNA (Sytox green, green) and NE (red). Nuclei were labeled by Hoechst 33342 (blue). Scale bar, 50 µm. (B) The relative fluorescence intensity of Sytox Green was used to determine the ET release. All the data are shown as the mean ± SEM (n = 4). One-way ANOVA with Dunnett’s test for multiple comparisons was used to test statistical significance (****, P < 0.0001; ns, not significant compared with the control group).

**
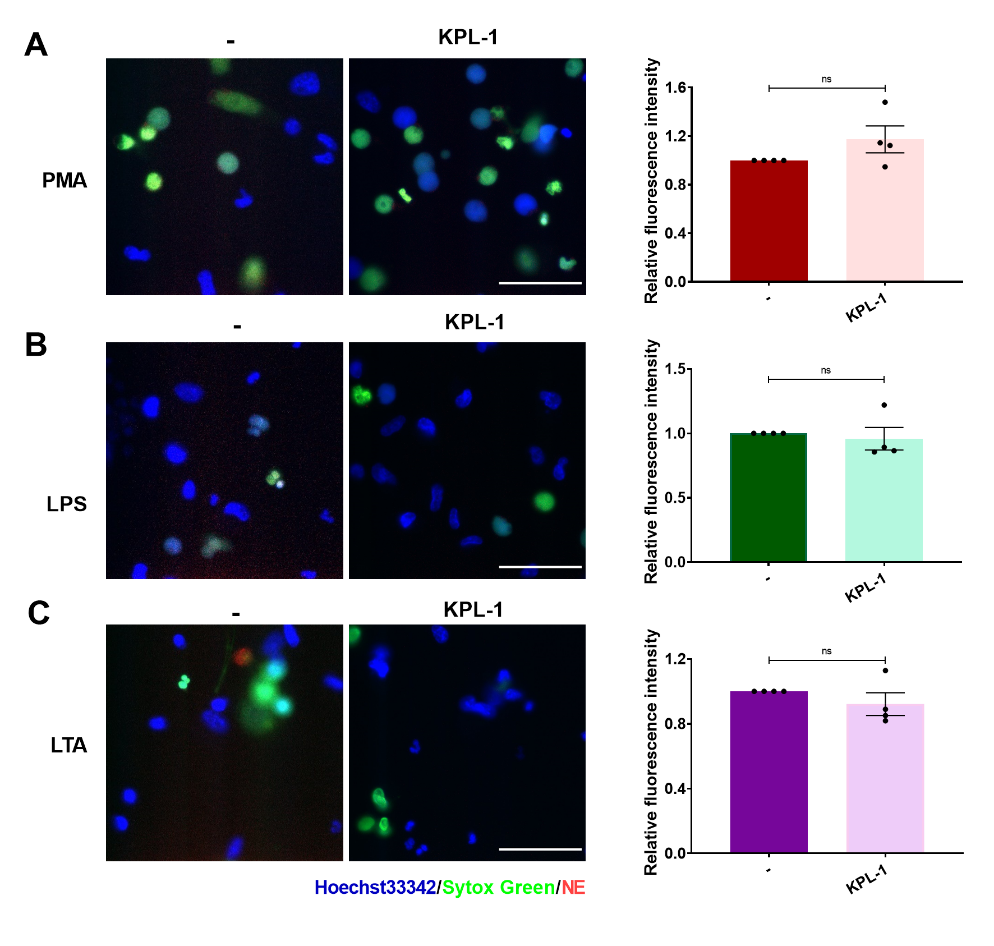
**

**Fig S2. PSGL-1 blockade did not affect NET release by neutrophils attached to HUVECs.** Neutrophils were pretreated with KPL-1(20 μg/mL, anti-PSGL-1 blocking Ab) for 30 min before incubation with HUVECs. (A-C) NETs from PMA- (100 ng/mL), LPS- (5 μg/mL), or LTA- (40 μg/mL) stimulated cells were visualized by staining DNA (Sytox green, green) and NE (red). Nuclei were labeled by Hoechst 33342 (blue). Scale bar, 50 µm. Relative fluorescence intensity of Sytox Green from the same experiment in response to PMA (A), LPS (B), and LTA (C) were shown. All the data are shown as the mean ± SEM (n = 4). Two-tailed Student’s t-test was used to test statistical significance (ns, not significant compared with the control group).

**
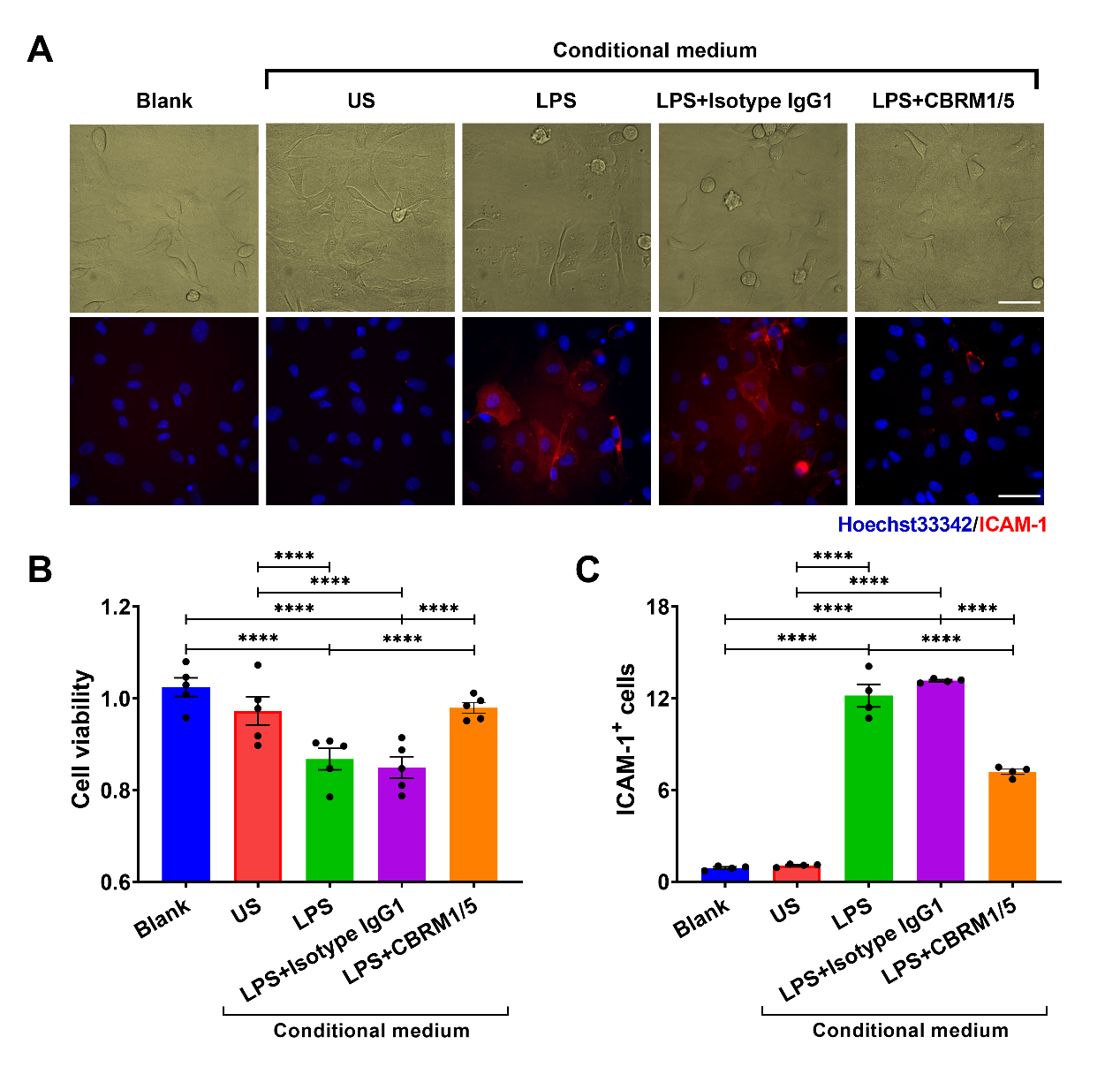
**

**Fig S3. Blocking Mac-1 mitigated NET-induced HUVEC injury.** (A) Representative bright image and immunofluorescence staining of the ICAM-1 in Blank control and conditional medium-treated HUVECs. Scale bar: 50 μm. (B) The cell viability of HUVECs treated with NETs. (C) The ICAM-1 positive HUVECs with treatments was measured by flow cytometry. All the data are shown as the mean ± SEM (n = 5). One-way ANOVA with Dunnett’s test for multiple comparisons was used to test statistical significance (****, P < 0.0001).

**
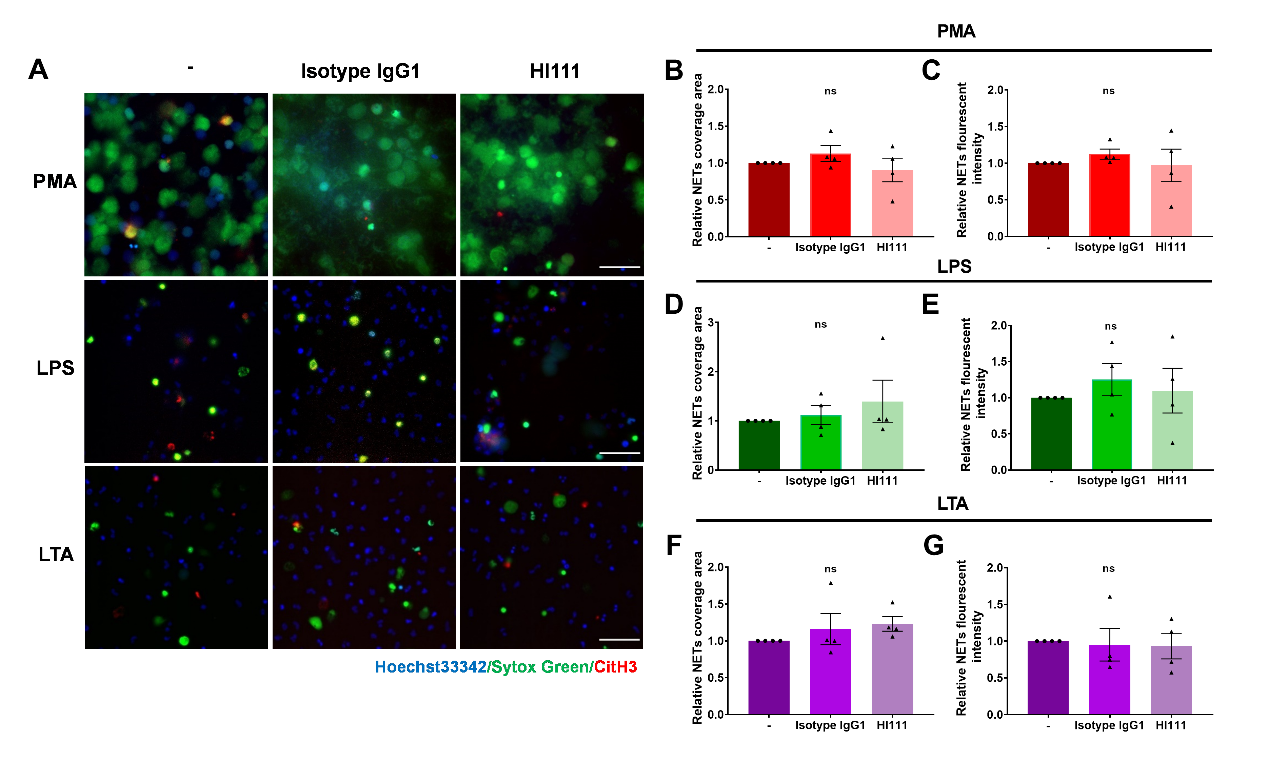
**

**Fig S4. LFA-1 is dispensable for NET formation in response to PMA, LPS, and LTA.** Neutrophils were pretreated with the anti-LFA-1 antibody HI111, or the isotype IgG and NET formation was induced by PMA, LPS, and LTA for 4 h at 37°C. (A) NETs were visualized by staining DNA (Sytox green, green) and CitH3 (red). Nuclei were labeled by Hoechst 33342 (blue). Scale bar, 50 μm. (B, D, F) The relative NETs coverage area induced by PMA (B), LPS (D), and LTA (F) after blocking LFA-1. (C, E, G) Relative NETs fluorescence intensity of NETs induced by PMA (C), LPS (E), and LTA (G) of the same experiment in (A) were presented. All the data are shown as the mean ± SEM (n = 4). One-way ANOVA with Dunnett’s test for multiple comparisons was used to test statistical significance (ns, not significant compared with the control group).

**
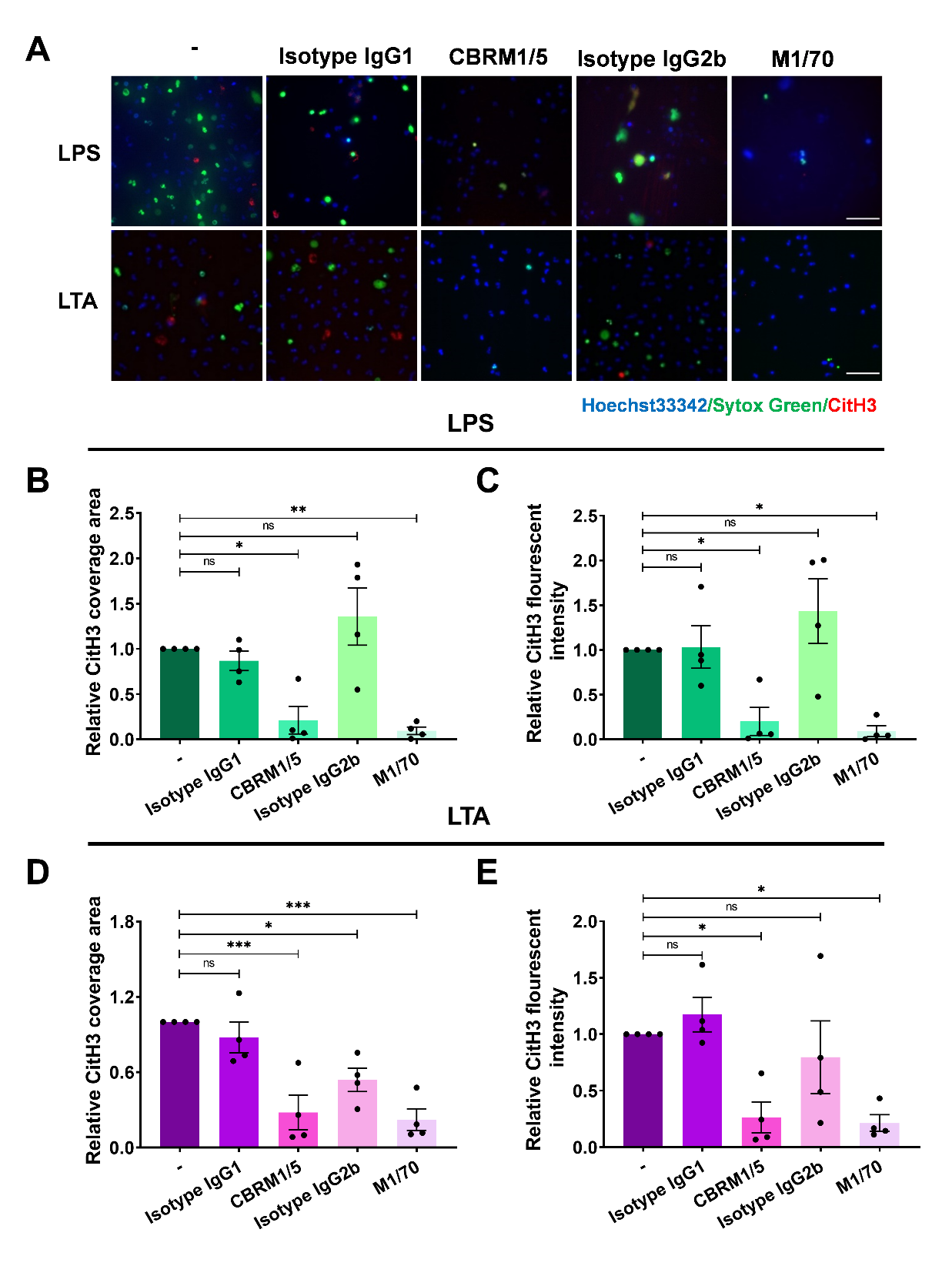
**

**Fig S5. Mac-1 induces the citrullination of histone H3 in response to LPS and LTA.** Neutrophils pretreated with anti-Mac-1 antibodies M1/70, CBRM1/5, or isotype IgG, or not were stimulated by LPS and LTA. (A) NETs were visualized by staining DNA (Sytox green, green) and CitH3 (red). Nuclei were labeled by Hoechst 33342 (blue). Scale bar, 50 μm. (B-E) In response to LPS (B, C) and LTA (D, E), relative CitH3 coverage area and fluorescence intensity after blocking Mac-1 were shown. All the data are shown as the mean ± SEM (n $=$ 4). One-way ANOVA with Dunnett’s test for multiple comparisons was used to test statistical significance (*, P < 0.05; **, P < 0.01; ***, P < 0.001; ****, P < 0.0001; ns, not significant compared with the control group).

**
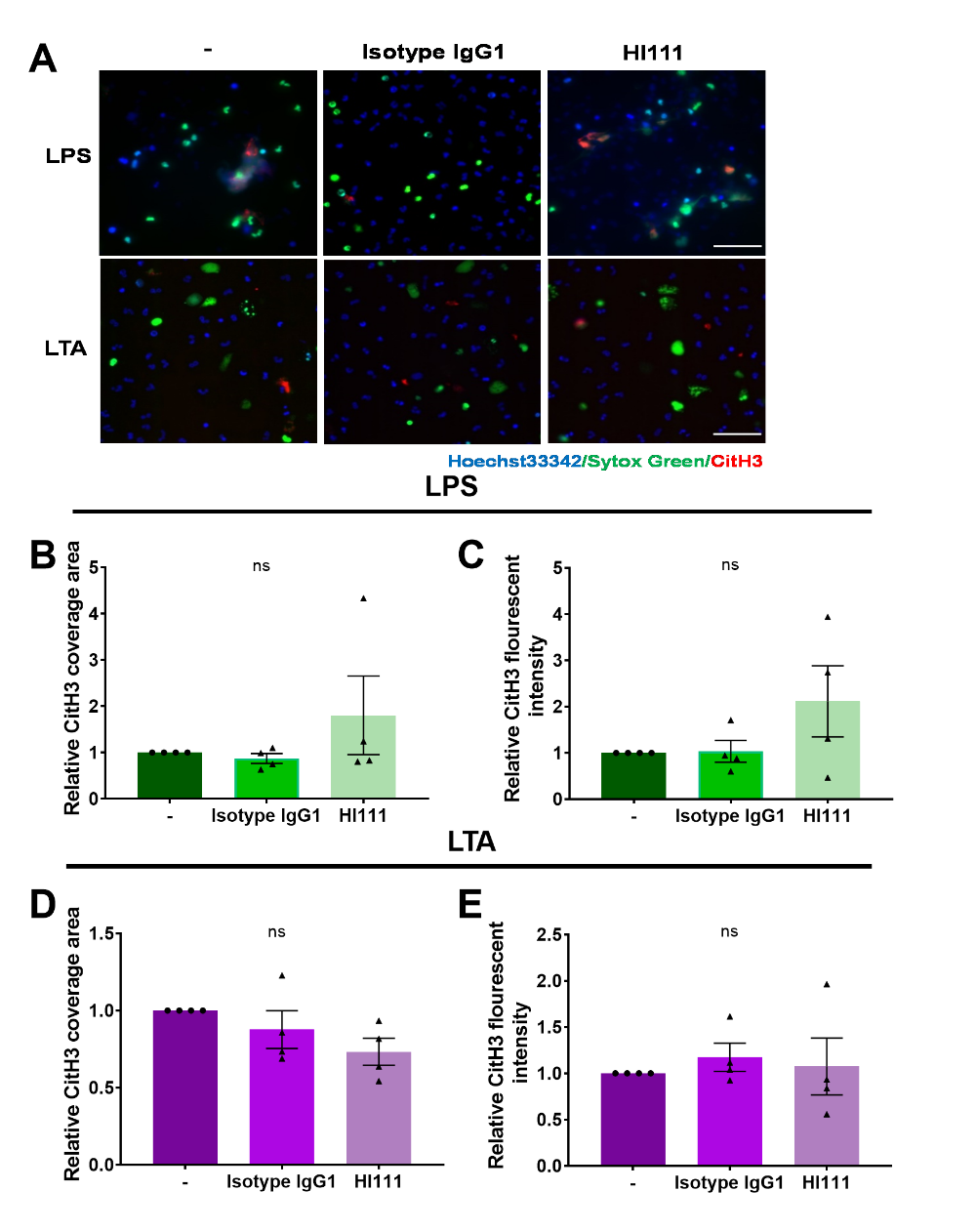
**

**Fig S6. LPS- or LTA-induced citrullination of histone H3 is independent of LFA-1.** Neutrophils pretreated with anti-LFA-1 antibody, HI111, or not were stimulated with LPS and LTA. (A) NETs were visualized by staining DNA (Sytox green, green) and CitH3 (red). Nuclei were labeled by Hoechst 33342 (blue). Scale bar, 50 μm. (B- E) In response to LPS (B, C) and LTA (D, E), relative CitH3 coverage area and fluorescence intensity after blocking LFA-1 were shown. All the data are shown as the mean ± SEM (n = 4). One-way ANOVA with Dunnett’s test for multiple comparisons was used to test statistical significance (ns, not significant compared with the control group).

**
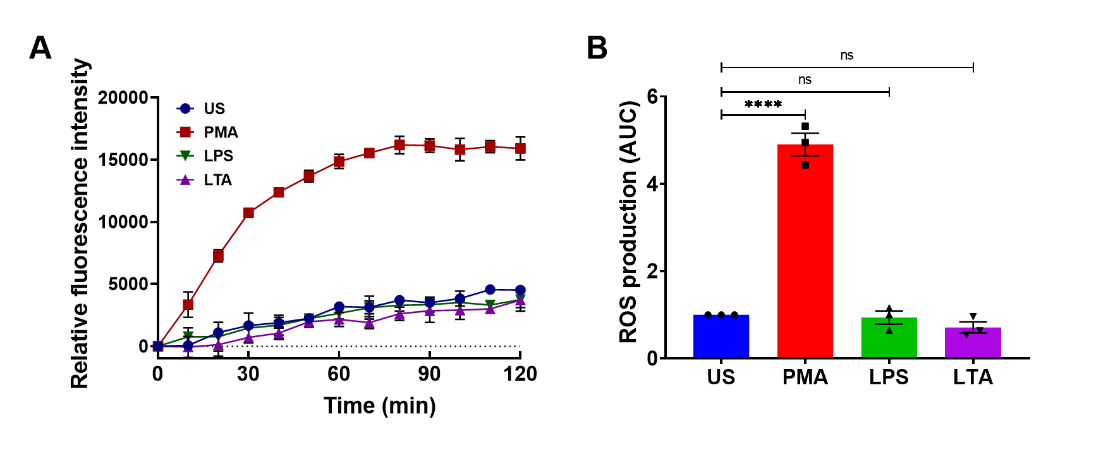
**

**Fig S7. LPS or LTA fails to induce ROS production.** Intracellular ROS traces were illustrated in real-time by CM-H_2_DCFDA-fluorescence (A) and the area under the curve (AUC) was calculated relative to the US group (B). All the data are shown as the mean ± SEM (n = 3). One-way ANOVA with Dunnett’s test for multiple comparisons was used to test statistical significance (****, *p* < 0.0001; ns, not significant compared with the control group).


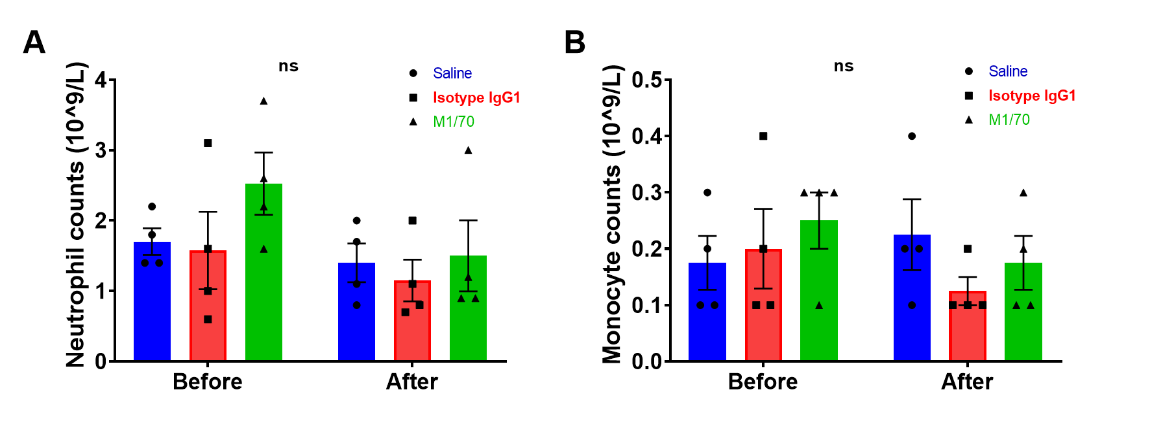


**Fig S8. The administration of M1/70 did not affect the cell count of the mice.** The counts of neutrophils (A) and monocytes (B) in mice before and after the administration of reagents. All the data are shown as the mean ± SEM (n =4). Two-way ANOVA with Sidak’s test for multiple comparisons was used to test statistical significance. ns, not significant.


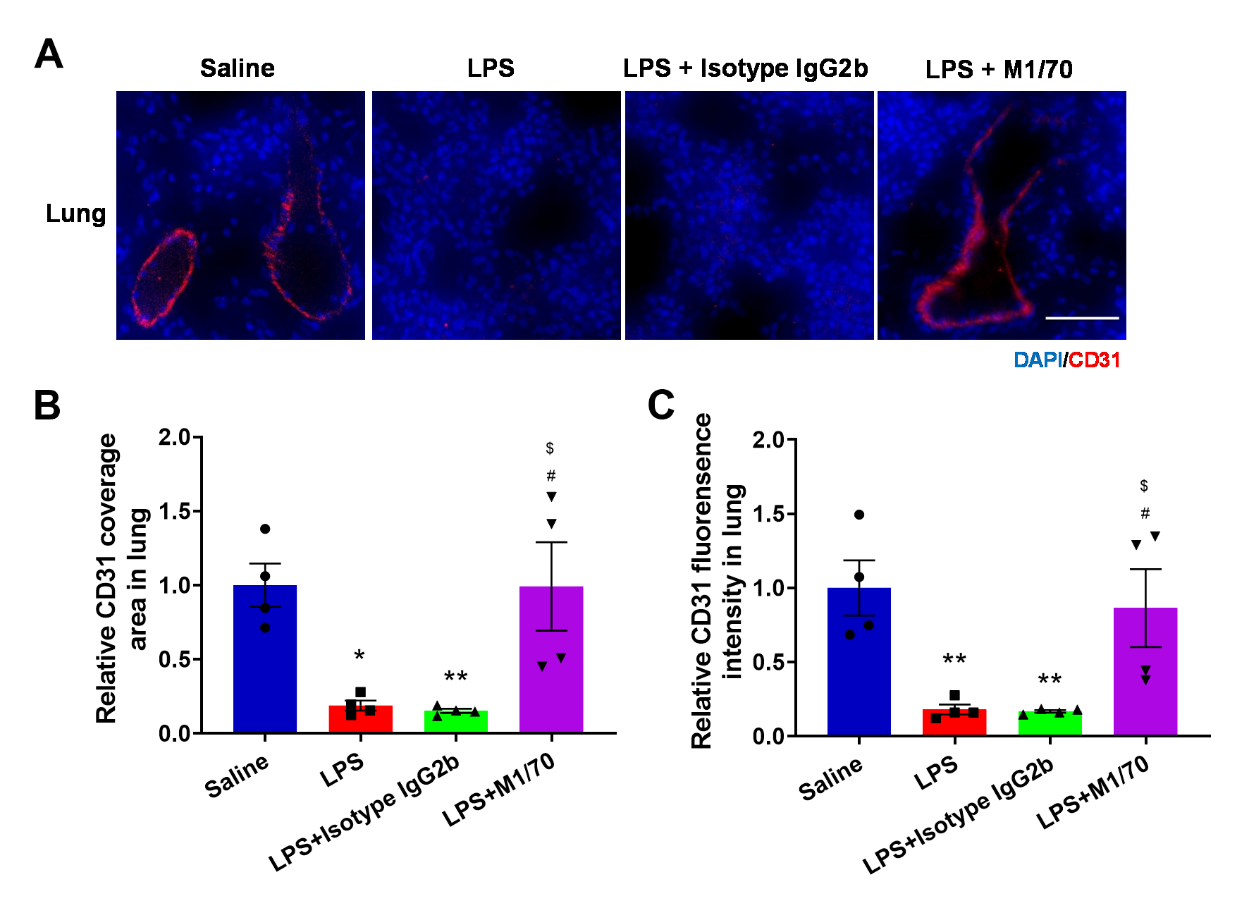


**Fig S9. Blocking Mac-1 reduced lung endothelium injury.** (A) Representative images of CD31 (red) stained in the lung tissues, nuclei with DAPI (blue). Scale bar, 50 μm. (B-C) Quantification of relative CD31 coverage area (B) and fluorescence intensity (C) in the lungs. Data represented as the mean ± SEM (n =4). One-way ANOVA with Dunnett’s test for multiple comparisons was used to test statistical significance (* p < 0.05, ** p < 0.01 vs. Saline, # p < 0.05 vs. LPS, $ p < 0.05 vs. LPS + Isotype IgG 2b).


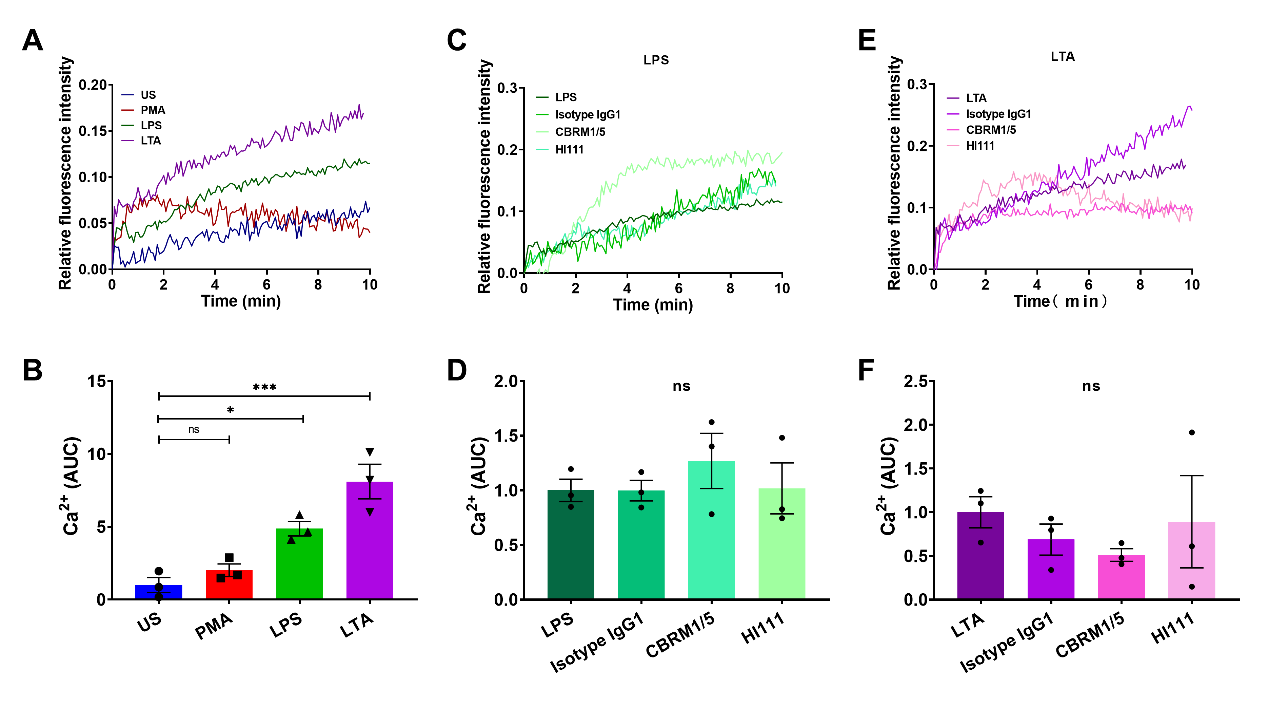


**Fig S10. Intracellular Ca^2+^ signals in response to PMA, LPS, or LTA.** The typical fluorescence intensity of calcium flux normalized by Hochest 33342 was presented in (A), and the area under the curve (AUC) was calculated relative to the US group (B). (C-E) Neutrophils that pre-incubated with inhibitors targeted Mac-1 before exposure to LPS (C, D) and LTA (E, F) respectively. All the data are shown as the mean ± SEM (n = 3). One-way ANOVA with Dunnett’s test for multiple comparisons was used to test statistical significance (*, P < 0.05; **, P < 0.01; ns, not significant compared with the control group).

**3 Legends for Videos**

**Video S1. Live-cell video of ICAM-1-mediated NET formation without stimulation.** Neutrophils on ICAM-1-coated glass surface without any stimulation were recorded by fluorescence microscope at 5 min intervals for 4 h. Hoechst 33342 (blue) and Sytox green (green) were used to label nuclei and extracellular DNA respectively. Scale bar, 50 μm.

**Video S2. Live-cell video of ICAM-1-mediated NET formation upon PMA stimulation.** Neutrophils on ICAM-1-coated glass surface were stimulated with PMA for 4 h and recorded by fluorescence microscope at 5 min intervals. Hoechst 33342 (blue) and Sytox green (green) were used to label nuclei and extracellular DNA respectively. Scale bar, 50 μm.

**Video S3. Live-cell video of ICAM-1-mediated NET formation upon LPS stimulation.** Neutrophils on ICAM-1-coated glass surface were stimulated with LPS for 4 h and recorded by fluorescence microscope at 5 min intervals. Hoechst 33342 (blue) and Sytox green (green) were used to label nuclei and extracellular DNA respectively. Scale bar, 50 μm.

**Video S4. Live-cell video of ICAM-1-mediated NET formation upon LTA stimulation.** Neutrophils on ICAM-1-coated glass surface were stimulated with LTA for 4 h and recorded by fluorescence microscope at 5 min intervals. Hoechst 33342 (blue) and Sytox green (green) were used to label nuclei and extracellular DNA respectively. Scale bar, 50 μm.

**Video S5. Live-cell video of neutrophils stimulated by PMA to form NETs after Mac-1 inhibition.** Neutrophils were pretreated with the anti-Mac-1 antibody CBRM1/5 and NET formation was induced by PMA for 4 h at 37°C. Hoechst 33342 (blue) and Sytox green (green) were used to label nuclei and extracellular DNA respectively. Scale bar, 50 μm.

**Video S6. Live-cell video of neutrophils stimulated by LPS to form NETs after Mac-1 inhibition.** Neutrophils were pretreated with the anti-Mac-1 antibody CBRM1/5 and NET formation was induced by LPS for 4 h at 37°C. Hoechst 33342 (blue) and Sytox green (green) were used to label nuclei and extracellular DNA respectively. Scale bar, 50 μm.

**Video S7. Live-cell video of neutrophils stimulated by LTA to form NETs after Mac-1 inhibition.** Neutrophils were pretreated with the anti-Mac-1 antibody CBRM1/5 and NET formation was induced by LTA for 4 h at 37°C. Hoechst 33342 (blue) and Sytox green (green) were used to label nuclei and extracellular DNA respectively. Scale bar, 50 μm.
